# The leaf idioblastome of the medicinal plant *Catharanthus roseus* is associated with stress resistance and alkaloid metabolism

**DOI:** 10.1093/jxb/erad374

**Published:** 2023-10-07

**Authors:** Joana G Guedes, Rogério Ribeiro, Inês Carqueijeiro, Ana Luísa Guimarães, Cláudia Bispo, John Archer, Herlander Azevedo, Nuno A Fonseca, Mariana Sottomayor

**Affiliations:** CIBIO, Centro de Investigação em Biodiversidade e Recursos Genéticos, InBIO Laboratório Associado, Campus de Vairão, Universidade do Porto, 4485-661 Vairão, Portugal; BIOPOLIS Program in Genomics, Biodiversity and Land Planning, CIBIO, Campus de Vairão, 4485-661 Vairão, Portugal; Programa Doutoral em Biologia Molecular e Celular (MCbiology), Instituto de Ciências Biomédicas Abel Salazar (ICBAS), Universidade do Porto, 4050-313 Porto, Portugal; CIBIO, Centro de Investigação em Biodiversidade e Recursos Genéticos, InBIO Laboratório Associado, Campus de Vairão, Universidade do Porto, 4485-661 Vairão, Portugal; BIOPOLIS Program in Genomics, Biodiversity and Land Planning, CIBIO, Campus de Vairão, 4485-661 Vairão, Portugal; Departamento de Biologia, Faculdade de Ciências da Universidade do Porto, 4169-007 Porto, Portugal; CIBIO, Centro de Investigação em Biodiversidade e Recursos Genéticos, InBIO Laboratório Associado, Campus de Vairão, Universidade do Porto, 4485-661 Vairão, Portugal; Departamento de Biologia, Faculdade de Ciências da Universidade do Porto, 4169-007 Porto, Portugal; Instituto Gulbenkian de Ciência, Rua da Quinta Grande 6, 2780-156 Oeiras, Portugal; CIBIO, Centro de Investigação em Biodiversidade e Recursos Genéticos, InBIO Laboratório Associado, Campus de Vairão, Universidade do Porto, 4485-661 Vairão, Portugal; BIOPOLIS Program in Genomics, Biodiversity and Land Planning, CIBIO, Campus de Vairão, 4485-661 Vairão, Portugal; CIBIO, Centro de Investigação em Biodiversidade e Recursos Genéticos, InBIO Laboratório Associado, Campus de Vairão, Universidade do Porto, 4485-661 Vairão, Portugal; BIOPOLIS Program in Genomics, Biodiversity and Land Planning, CIBIO, Campus de Vairão, 4485-661 Vairão, Portugal; Departamento de Biologia, Faculdade de Ciências da Universidade do Porto, 4169-007 Porto, Portugal; CIBIO, Centro de Investigação em Biodiversidade e Recursos Genéticos, InBIO Laboratório Associado, Campus de Vairão, Universidade do Porto, 4485-661 Vairão, Portugal; BIOPOLIS Program in Genomics, Biodiversity and Land Planning, CIBIO, Campus de Vairão, 4485-661 Vairão, Portugal; CIBIO, Centro de Investigação em Biodiversidade e Recursos Genéticos, InBIO Laboratório Associado, Campus de Vairão, Universidade do Porto, 4485-661 Vairão, Portugal; BIOPOLIS Program in Genomics, Biodiversity and Land Planning, CIBIO, Campus de Vairão, 4485-661 Vairão, Portugal; Departamento de Biologia, Faculdade de Ciências da Universidade do Porto, 4169-007 Porto, Portugal; University of Malaga, Spain

**Keywords:** Abiotic stress, anticancer drugs, biotic stress, *Catharanthus roseus*, fluorescence-activated cell sorting (FACS), idioblasts, monoterpenoid indole alkaloids, protoplasts, transcriptome, vinblastine

## Abstract

*Catharanthus roseus* leaves produce a range of monoterpenoid indole alkaloids (MIAs) that include low levels of the anticancer drugs vinblastine and vincristine. The MIA pathway displays a complex architecture spanning different subcellular and cell type localizations, and is under complex regulation. As a result, the development of strategies to increase the levels of the anticancer MIAs has remained elusive. The pathway involves mesophyll specialized idioblasts where the late unsolved biosynthetic steps are thought to occur. Here, protoplasts of *C. roseus* leaf idioblasts were isolated by fluorescence-activated cell sorting, and their differential alkaloid and transcriptomic profiles were characterized. This involved the assembly of an improved *C. roseus* transcriptome from short- and long-read data, IDIO+. It was observed that *C. roseus* mesophyll idioblasts possess a distinctive transcriptomic profile associated with protection against biotic and abiotic stresses, and indicative that this cell type is a carbon sink, in contrast to surrounding mesophyll cells. Moreover, it is shown that idioblasts are a hotspot of alkaloid accumulation, suggesting that their transcriptome may hold the key to the in-depth understanding of the MIA pathway and the success of strategies leading to higher levels of the anticancer drugs.

## Teaser

*Catharanthus* mesophyll idioblasts are a hotspot of alkaloid accumulation. The idioblast transcriptome is associated with stress responses and provides a roadmap towards the increase of anticancer alkaloid levels.

## Introduction

Understanding plant specialized metabolism is central to decipher plant defence strategies, as well as to fully exploit its diverse human applications, often hindered by low levels of metabolites and limited availability of plant material. The advent of high-throughput sequencing has provided a way to tackle these hurdles, by enabling the thorough transcriptomic profiling of conditions, organs, tissues, or cell types associated with specific metabolic profiles, thus generating unprecedented insights into the genetic determinants of the architecture and dynamics of specialized metabolism (Dugé de Bernonville *et al.*, 2020; Courdavault *et al*., 2021).

**Fig. 1.**
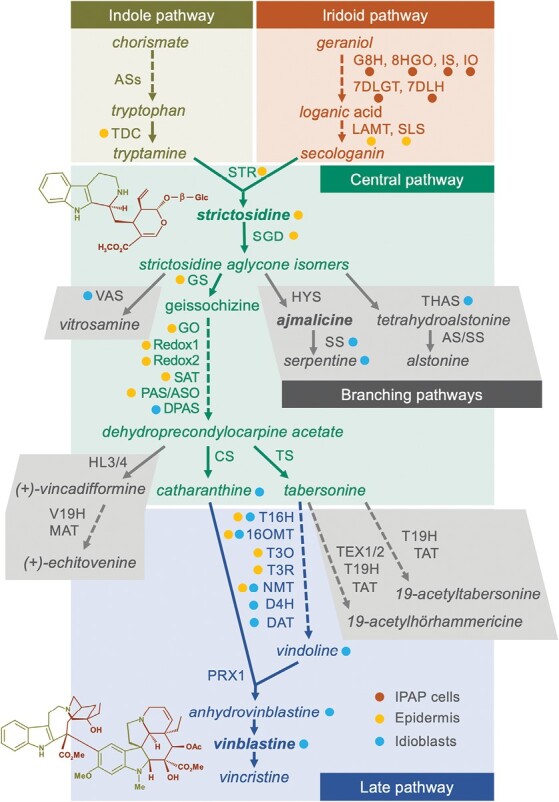
The MIA pathway in *C. roseus.* The indole and iridioid monoterpenoid pathways generate the indole precursor tryptamine and the monoterpenoid precursor secologanin, which condensate to yield strictosidine, the first MIA and the precursor of all MIA scaffolds. The central MIA pathway generates catharanthine and tabsersonine, and is thought to occur in the epidermis. The late MIA pathway involves the conversion of tabersonine into vindoline, and the steps leading to the anticancer alkaloids vinblastine and vincristine. Idioblast localization is represented by blue dots and is based on the results of this study. Epidermal and IPAP (internal phloem-associated parenchyma) localization is represented by yellow and red-orange dots, respectively, and has been previously published, corresponding to transcript localization by *in situ* analysis or presence in an epidermal RNA library, complemented in a few cases with protein localization using antibodies (St-Pierre *et al.*, 1999; Irmler *et al.*, 2000; Burlat *et al.*, 2004; Murata *et al.*, 2008; Guirimand *et al.*, 2011a, b; Geu-Flores *et al.*, 2012; Miettinen *et al.*, 2014; Qu *et al.*, 2015, 2018a, b, 2019). Structural formulae correspond to strictosidine (above) and vinblastine (below), with colours representing the pathway origin of each part of the backbone. The indole pathway: ASs, anthranilate synthase; TDC, tryptophan decarboxylase. The iridoid pathway: G8H, geraniol 8-hydroxylase; 8HGO, 8-hydroxygeraniol oxidoreductase; IS, iridoid synthase; IO, iridoid oxidase; 7DLGT, 7-deoxyloganetic acid glucosyltransferase; 7DLH, 7-deoxyloganic acid hydroxylase; LAMT, loganic acid *O*-methyltransferase; SLS, secologanin synthase. The central pathway: STR, strictosidine synthase; SGD, strictosidine-β-D-glucosidase; GS, geissoschizine synthase; GO, geissoschizine oxidase; SAT, stemmadenine-*O*-acetyltransferase; PAS/ASO, precondylocarpine acetate synthase/*O*-acetylstemmadenine oxidase; DPAS, dihydroprecondylocarpine synthase; CS, catharanthine synthase; TS, tabersonine synthase. The late pathway: T16H, tabersonine 16-hydroxylase; 16OMT, tabersonine 16-*O*-methyltransferase; T3O, tabersonine 3-oxygenase; T3R, tabersonine 3-reductase; NMT, 3-hydroxy-16-methoxy-2,3-dihydrotabersonine *N*-methyltransferase; D4H, desacetoxyvindoline-4-hydroxylase; DAT, deacetylvindoline-4-*O*-acetyltransferase; PRX1, class III peroxidase 1. Branching pathways: VAS, vitrosamine synthase; HYS, heteroyohimbine synthase; THAS, tetrahydroalstonine synthase; AS, alstonine synthase; SS, serpentine synthase; HL, hydrolase; V19H, vincadifformine-19-hydroxylase; MAT, minovincinine-19-*O*-acetyltransferase; TEX, tabersonine epoxidase; T19H, tabersonine 19-hydroxylase; TAT, 19-hydroxytabersonine 19-*O*-acetyltransferase.

*Catharanthus roseus* (L.) G. Don (*Apocynaceae*) is the source of the low level anticancer drugs vinblastine and vincristine, and of the antihypertensive drug ajmalicine (Fig. 1). These drugs are monoterpenoid indole alkaloids (MIAs) and, despite intense research, the MIA pathway still hides several blindspots and remains a challenge regarding the low levels of vinblastine and vincristine. The biosynthesis of the dimeric vinblastine from the basic precursors tryptophan (indole moiety precursor) and geranyl pyrophosphate (monoterpenoid moiety precursor) involves nearly 30 enzymatic steps (Fig. 1). The pathway has been elucidated up to the coupling of the vinblastine monomeric building blocks, vindoline and catharanthine, into α-3ʹ,4ʹ-anhydrovinblastine (anhydrovinblastine) (Costa *et al.*, 2008; Dugé de Bernonville *et al*., 2015a; Kulagina *et al.*, 2022). However, the conversion of anhydrovinblastine into the extremely low-level vinblastine and vincristine remains elusive.

MIA biosynthesis and accumulation in *C. roseus* is modulated by jasmonates and is under multilayer transcriptional regulation that has been partially deciphered, including the characterization of several transcription factors inducing and repressing the early pathways (Patra *et al.*, 2018; Schweizer *et al.*, 2018; Colinas *et al.*, 2021). Promising results were obtained recently with the characterization of the transcription factors ethylene responsive factor 5 (ERF5) and GATA-binding protein 1 (GATA1), which, for the first time, enabled up-regulation of the late MIA pathway genes and alkaloids (Liu *et al.*, 2019; Pan *et al.*, 2019). Nevertheless, the transcriptional regulatory network of MIA biosynthesis is far from being fully understood.

The MIA pathway displays a surprisingly complex spatial architecture, spanning five subcellular compartments throughout four different cell types in *C. roseus* leaves, the organ where the dimeric anticancer drugs vinblastine and vincristine are specifically produced and accumulated (Courdavault *et al.*, 2014; De Luca *et al.*, 2014; Dugé de Bernonville *et al*., 2015a; Kulagina *et al.*, 2022). The monoterpenoid moiety pathway (Fig. 1) starts in the chloroplasts and cytosol of internal phloem-associated parenchyma (IPAP) leaf cells, after which it proceeds to the leaf epidermis, where the monoterpenoid and indole final precursors, secologanin and tryptamine, are generated in the cytosol (Burlat *et al.*, 2004; Miettinen *et al.*, 2014). Secologanin and tryptamine are condensed inside the vacuole of epidermal cells by strictosidine synthase (STR) to yield strictosidine, the first MIA of the pathway and the central precursor of all MIA scaffolds (Fig. 1) (Guirimand *et al.*, 2011a). Many of the subsequent reactions appear to proceed in the epidermis, a localization that has also been observed for other alkaloid pathways and is consistent with the assumed defence role of these compounds (Guirimand *et al.*, 2011b; Qu *et al.*, 2019). The final steps of vindoline biosynthesis were shown to be specifically expressed in leaf laticifers and in specialized mesophyll cells—the idioblasts—both of which have also been shown to constitute leaf alkaloid accumulation targets (Yoder and Mahlberg, 1976; Mersey and Cutler, 1986; St- Pierre *et al.*, 1999; Carqueijeiro *et al.*, 2016; Yamamoto *et al.*, 2019). All multiple intra- and intercellular transmembrane transport events involved in the MIA pathway’s architecture are putative rate-limiting steps of MIA metabolic fluxes. However, so far, only a few of those events have been (partially) characterized, including: vacuolar accumulation of vindoline, catharanthine, and anhydrovinblastine by a H^+^-antiport system; efflux of catharanthine to the leaf surface by an ATP-binding cassette G (ABCG) transporter; vacuolar export of strictosidine by a nitrate/peptide family (NPF) transporter; and the involvement of three NPF transporters in the transport of iridoid intermediates (Carqueijeiro *et al.*, 2013; Yu and De Luca, 2013; Larsen *et al.*, 2017; Payne *et al.*, 2017).

Leaf idioblasts, whose vacuoles are likely a final alkaloid accumulation target, are potentially the home of the important vinblastine/vincristine biosynthetic steps and of transmembrane transport events with a high impact in the metabolic flux of the MIA pathway. In this study, *C. roseus* leaf idioblast protoplasts were isolated using a fluorescence-activated cell sorting (FACS) methodology (Carqueijeiro *et al.*, 2016), and the characterization of the differential alkaloid and transcriptomic profile of this cell type was performed. The sequence data obtained enabled the generation of a comprehensive transcriptome, IDIO+, which represents a new valuable tool for the species. It is shown that the *C. roseus* mesophyll idioblasts possess a unique transcriptomic profile, deeply implicated in protection against biotic and abiotic stresses, and displaying low expression of genes involved in photosynthesis, in comparison with common mesophyll cells. It is also shown that idioblasts accumulate much higher levels of MIAs than other leaf cells, suggesting that their transcriptome information may be decisive to the understanding of the vinblastine/vincristine pathway towards manipulation of *C. roseus* for higher levels of those anticancer drugs.

## Materials and methods

### Plant material

*Catharanthus roseus* (L.) G. Don ‘Little Bright Eye’ plants were grown at 25 °C in a growth chamber under a 16 h/8 h photoperiod, using white fluorescent light with a photon medium intensity of 70 μmol m^–2^ s^–1^. Seeds were acquired from B&T World Seeds (France).

For the indoor/outdoor experiment, one group of *C. roseus* mature flowering plants were kept in the growth chamber, while an equivalent group was transferred outdoors, under natural environmental conditions and exposed to direct sunlight during ~6 months. Leaves of the first, second, third, and fourth pair (counting from the shoot apical meristem) of three plants from each set were collected at around 14.00 h, at the beginning of September, on a hot (>30 °C) sunny day. After the removal of central veins, leaves were macerated with liquid nitrogen and part was stored at –80 °C for RNA extraction, while the remainder was freeze-dried and stored at –20 °C for alkaloid extraction.

### Isolation of leaf protoplasts and FACS

Six-month-old plants were used for protoplast isolation and three biological replicates were performed. Each replicate contained 1.5–2 g of fully developed healthy young leaves from the second to the fourth pair (counting from the shoot apex), collected from three mutually exclusive groups of 3–5 plants. Protoplast isolation was performed as previously described in Carqueijeiro *et al.* (2013). The FACS methodology is described in Carqueijeiro *et al.* (2016) and Guedes *et al.* (2022). Matching leaf samples were also collected and processed as described in the plant material section.

### Alkaloid extraction and analysis by HPLC-MS

Fresh material was freeze-dried immediately after being collected. Leaf samples contained 70–180 mg FW. The number of cells used for each sorted sample was 200 000 for total protoplasts, 300 000 for mesophyll protoplasts, and 30 000 for idioblast protoplasts. Alkaloids were extracted by adding 150 µl or 500 µl of HPLC grade methanol to cell or leaf samples, respectively. Papaverine was added at this stage as an internal control: 17.5 µg or 35 µg to cell or leaf samples, respectively, using a 2.5 mg ml^–1^ methanolic solution. Samples were resuspended and extracted by vortexing during 2 min, followed by sonication for 30 min, and centrifugation at 10 000 *g* for 10 min. Supernatants were filtered and analysed by HPLC-electrospray ionization-tandem MS as described in Carqueijeiro *et al.* (2016). All alkaloid standards were obtained from Sigma Aldrich, except anhydrovinblastine, which was obtained from Pierre Fabre. Retention times (RTs) and masses were as follows: serpentine, RT=8.03 min, *m/z* 349.15; papaverine, RT=10.98 min, *m/z* 340.15; vindoline, RT=19.30 min, *m/z* 457.23; vincristine, RT=26.12 min, *m/z* 825.41; catharanthine, RT=29.00 min, *m/z* 337.19; ajmalicine, RT=34.04 min, *m/z* 353.19; vinblastine, RT=38.63 min, *m/z* 811.43; anhydrovinblastine, RT=49.20 min, *m/z* 793.42. For calculation of cell millimolar concentration, a medium diameter of *n*=132 protoplasts (including idioblasts and common mesophyll cells) was measured by ImageJ software and the protoplast medium volume was calculated under the assumption that the protoplasts are spherical.

### RNA extraction, library preparation, and sequencing

RNA was extracted from paired samples of whole leaves, total protoplasts (200 000 cells), sorted idioblast protoplasts (50 000 cells), and sorted mesophyll protoplasts (120 000 cells). To purify RNA from the three protoplast populations, a solution of 1% (v/v) β-mercaptoethanol in RNeasy Lysis Buffer (Qiagen) was added in a volume ratio of 3.5:1 of cell suspension. Cells were disrupted by vigorous vortexing, flash-frozen in liquid nitrogen, and stored at –80 °C. RNA extraction was performed with the Rneasy Micro Kit (Qiagen) protocol in agreement with the manufacturer’s instructions. For whole leaf samples, processed as described in the plant material section, RNA was extracted with the Rneasy Plant Mini Kit (Qiagen) following the manufacturer’s instructions. All RNA extracts were precipitated with RNA-grade glycogen (Thermo Scientific), according to the manufacturer’s protocol. Due to RNA degradation, one leaf sample was discarded. For the FACS experiment, libraries were prepared using the Smart-seq2 kit following the manufacturer’s protocol (Picelli *et al.*, 2013). For the indoor/outdoor experiment, libraries were prepared using the TruSeq RNA Library Prep Kit. Sequencing was performed with Illumina HiSeq1500 on pair-end mode (2 × 125 bp) for 250 cycles. Long-read libraries were prepared with Nanopore SQK-PCB109 kit following the manufacturer’s protocol. Sequencing was performed on single-end mode with a MinION sequencer using an R9.4.1 flow cell for 71 h.

### Data quality control and filtering

Illumina raw data were processed with Trimmomatic (version 0.39) (parameters: ILLUMINACLIP:2:30:10 LEADING:3 TRAILING:10 SLIDINGWINDOW:4:15 MINLEN:50) to remove adaptors and low-quality bases, and to filter reads with ≤50 bp (Bolger *et al.*, 2014). For Nanopore data, basecalling and demultiplexing were performed using the Guppy v4.0.14 [Oxford Nanopore Technologies (ONT) Oxford, UK] high accuracy model. All reads were oriented based on barcode sequences using Pychopper (version 2.4, https://github.com/Nanoporetech/pychopper). During this process, barcode sequences were trimmed, low-quality reads were removed, and reads with <50 bp were discarded. Both ‘oriented’ and ‘rescued’ reads were used for downstream analysis. Data quality and statistics were verified using the FastQC tool (version 0.19.11, https://www.bioinformatics.babraham.ac.uk/projects/fastqc/).

### Genome-guided transcriptome assembly and annotation

Following read filtering, all samples were mapped to the *C. roseus* genome v2 (Franke *et al.*, 2019; https://datadryad.org/stash/dataset/doi:10.5061/dryad.08vv50n). For Illumina data, reads were aligned using HISAT2 (version 2.2.0) with the ‘-dta’ option enabled (Kim *et al.*, 2019). Following this step, a transcriptome for each sample was assembled using Stringtie (version 2.1.4) setting the minimum isoform abundance per locus to 0.05 (Kovaka *et al.*, 2019). Nanopore long reads were mapped to the *C. roseus* genome v2 using minimap2 (version 2.15-r905 using parameters -ax splice -uf; Li, 2018), and the primary alignments were used for assembly with Stringtie (using parameters -rf -L).

The Mikado pipeline (version 2.1.0) was then used to merge Stringtie runs and remove redundant sequences (Venturini *et al.*, 2018). Several metrics related to each gene model were computed to aid in improving the final transcriptome reconstruction. Sequences were subjected to ORF calling using TransDecoder (version 5.5.0; Haas *et al.*, 2013) and to homology searches against the Swiss-Prot viridiplantea database using DIAMOND (version 2.0.6 using parameters -e 0.00001 -sensitive -max-target-seqs 50; Buchfink *et al.*, 2015). High-quality splicing junctions were obtained from the Illumina alignments using Portcullis (Mapleson *et al.*, 2018). The CDF97 transcriptome (Dugé de Bernonville *et al.*, 2015b) *C. roseus* complete genes downloaded from NCBI (accessed on 14 February 2021) were mapped to the genome v2 reference annotation using minimap2, and sequences >2500 bp containing two or more hits (>90% query coverage and >80% identity) were identified as putative chimeric reference gene models. The final set of transcripts was obtained using Mikado pick with the ‘plant.yaml’ scoring file, with the above-mentioned fusion gene models being penalized. The genome browser Jbrowser (Buels *et al.*, 2016) was used for visualization of resulting gene models, and manual curation was performed when necessary. GTF and FASTA files including all the mRNA gene models were generated (Supplementary Datasets S1, S2). Gffcompare (Pertea and Pertea, 2020) was used for identification of novel genes, by comparing the final transcriptome with the reference gene models (Supplementary Table S1). BUSCO (Benchmarking set of Universal Single-Copy Orthologs; version 5.0.0) was used to assess the completeness of the final transcriptome using the eudicots_db10 dataset as queries. The full IDIO+ transcriptome (TSA, transcriptome shotgun assembly), including coding, non-coding, and splicing variants, has been deposited at DDBJ/EMBL/GenBank under the accession GKIJ00000000. The version described herein is the first version, GKIJ0100000.

The functional annotation of coding protein sequences was performed using both the pannzer2 (Toronen *et al.*, 2018) web server (http://ekhidna2.biocenter.helsinki.fi/sanspanz/) and the InterproScan software (version 5.50-84.0) (Quevillon *et al.*, 2005) with default parameters (Supplementary Dataset S3). Only the representative transcript isoform for each gene (defined as the higher scoring Mikado transcript for each gene) was annotated. Gene Ontology (GO) terms were obtained from pannzer2 annotation. Enzymes, transporters, and transcription factors from the MIA pathway were annotated manually, with the GO annotation being retrieved from the Uniprot database.

### Quantification and exploratory analysis

Following mapping, quantification of short reads was obtained with featureCounts (version 2.0.1, parameters: -p -t exon -g gene_id) using the mRNA gene models gtf file (Supplementary Dataset S1). Transcripts per million (TPM) values were calculated using the formula: 10^6^×(reads_mapped_to_transcript/ transcript_length)/Sum (reads_mapped_to_transcript/ transcript_length), where reads_mapped_to_transcript is the total number of reads mapped to a gene and transcript length is the size of the gene as outputted by featureCounts. Principal component analysis (PCA) and hierarchical clustering based on sample correlation were performed on short-read data. Count data were imported to R, normalized using the DESeq2 package (version 1.28.1), and transformed using the rlog function (Love *et al.*, 2014). For the PCA, only the top 500 genes, in terms of variance, were considered. Spearman correlation was used for the hierarchical clustering and the figure was generated using the pheatmap package. The RNA-seq data have been deposited in NCBI’s Gene Expression Omnibus (Edgar *et al*., 2002) and are accessible through GEO Series accession number GSE217852 (https://www.ncbi.nlm.nih.gov/geo/query/acc.cgi?acc=GSE217852).

### Differential gene expression analysis

Differential gene expression (DGE) analysis was performed using the DESeq2 package (Soneson *et al.*, 2015). For the analysis, idioblast, mesophyll, and total protoplast samples, as well as indoor and outdoor plant samples from leaf pairs 1 and 4 (12 samples; short reads), were incorporated into the statistical model. Leaf samples from the FACS experiment were excluded from the model due to a higher within-group variance (Supplementary Fig. S1). Only genes with adjusted *P*-values ≤0.05 and absolute log_2_ fold change (FC) ≥1 were considered differentially expressed. The results of DGE are included in Supplementary Tables S2–S4. The TPM expression levels for all the samples used can be found in Supplementary Dataset S4.

### Weighted gene co-expression network analysis

Expression levels from mesophyll and idioblast protoplast samples as well as from leaf pairs 1 and 4 from plants growing indoors/outdoors were used to build a co-expression network using the R package WGCNA (version 1.69; Langfelder and Horvath, 2008). To filter genes with low expression levels, count data were first normalized into counts per million (CPM). Only genes with >1.5 CPM in at least six of the samples were considered for network construction. The filtered gene matrix with raw counts was then subjected to normalization using DESeq2 (Soneson *et al.*, 2015) and was subjected to variance-stabilizing transformation (Anders and Huber, 2010). For the signed network construction, an adjacency matrix was computed by raising to a power of 20 (soft threshold) the Pearson correlation between the expression profiles of every pair of genes (Supplementary Fig. S2). A topological overlap matrix was then computed based on the adjacency values and the network was constructed using the corresponding dissimilarity matrix. Gene modules were identified using the cutreeDynamic function, with the minimum module size set to 30. Resulting modules were clustered using a representative expression profile (the module ‘eigengene’), and correlated modules with a maximum height of 0.15 were merged. Enrichment analysis for each module was performed as described below using the network genes as the background annotation.

For module–trait correlation analysis, the measurements of catharanthine, vindoline, anhydrovinblastine, and vinblastine in idioblast and common mesophyll protoplast samples were converted from ng per cell into ng mg^–1^ DW. It was assumed that the alkaloid content of total protoplasts and leaves was roughly the same, and the average ratio between pair-wise leaf/protoplast samples was applied to idioblast and common mesophyll protoplast samples to estimate values in ng mg^–1^ DW. Module–trait correlation analysis was performed by computing the Pearson correlation between module eigengenes and alkaloid levels. Correlations with a false discovery rate- (FDR) adjusted *P*-value <0.05 were considered significant (Benjamini and Hochberg, 1995).

### Enrichment analysis

Enrichment analysis in GO terms was performed using the topGO package version 2.40 (Alexa *et al.*, 2006) for both up-regulated and down-regulated gene sets. The background annotation was defined as the full list of genes expressed in either idioblasts or common mesophyll protoplasts. For the analysis, GO terms with <5 annotated genes were excluded, to prevent statistical artefacts. The ‘weigth01’ algorithm was used during the analysis. Fisher’s exact test was used to calculate significance for all tests, with *P*-values ≤0.01 being considered as statistically significant.

### Phylogenetic analysis

For phylogenetic analysis, the dicots PLAZA portal (version 4.5; https://bioinformatics.psb.ugent.be/plaza/versions/plaza_v4_5_dicots/) was surveyed to obtain all protein sequences for each studied gene family, from the genomes of *Arabidopsis thaliana*, *Amborella trichopoda*, and *Oryza sativa* (Van Bel *et al.*, 2012). In addition, protein sequences of members with known functions in specialized metabolism, as concluded from a literature survey, were retrieved from GenBank. Protein sequence data were aligned with the *C. roseus* candidate sequences using MAFFT (version 7.475, default parameters, Katoh and Standley, 2013), and the most fitting evolutionary model was determined with SMS version 1.8.4 (Lefort *et al.*, 2017) using the BIC (Bayesian information criterion) as the selection criterion (Supplementary Table S5). Phylogenetic analyses were performed on the CIPRES portal (http://www.phylo.org/) (Miller *et al.*, 2011) using RaxML version 8.2.12 (Stamatakis, 2014), with 1000 rapid bootstraps. Resulting trees were visualized and edited using FigTree (version 1.4.4; http://tree.bio.ed.ac.uk/software/figtree/).

## Results

### Anticancer alkaloids are specifically accumulated in idioblasts

**Fig. 2.**
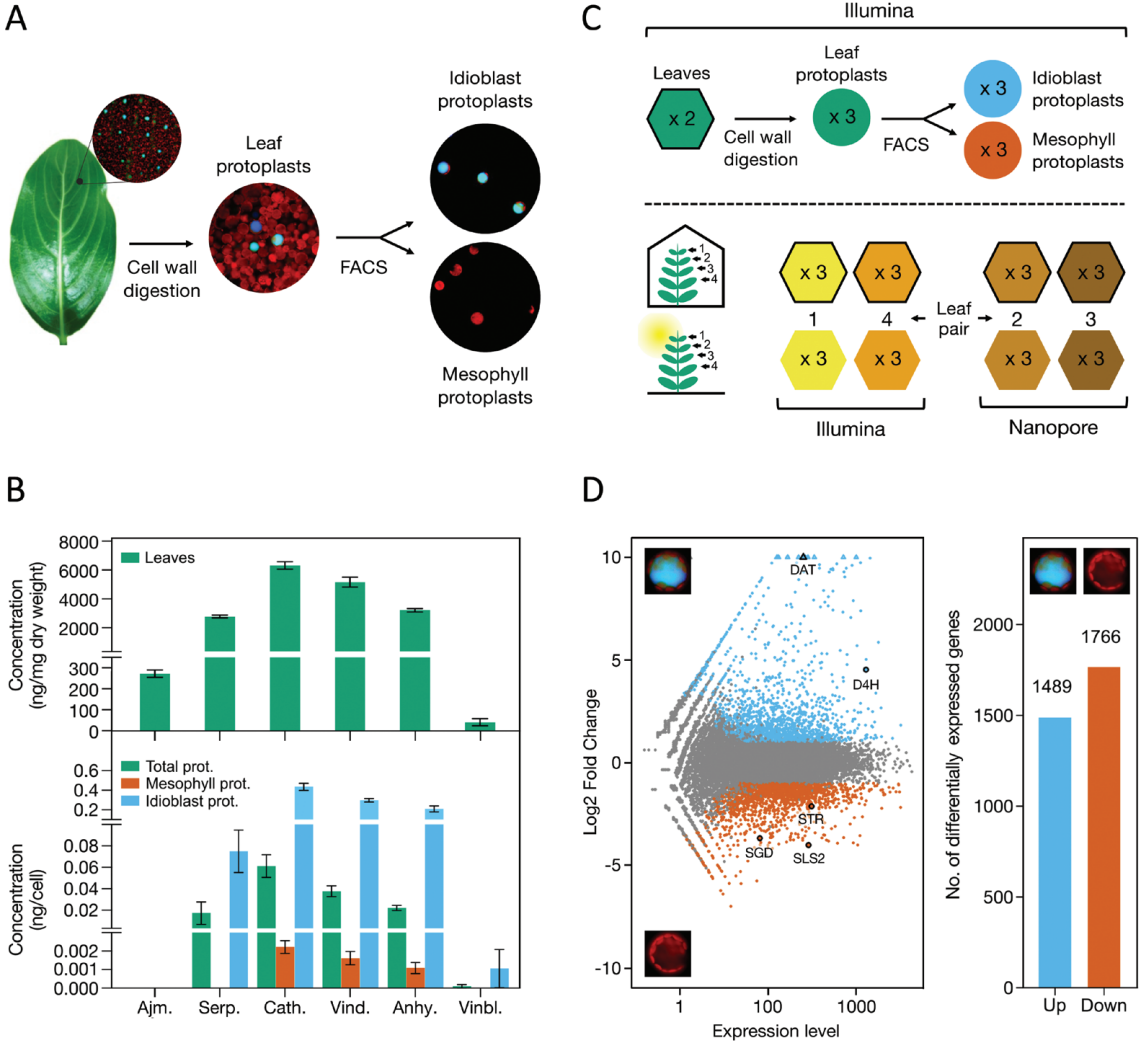
(A) Outline of the experimental setup used to generate populations of *C. roseus* leaf idioblast cells and common mesophyll cells. FACS, fluorescence-activated cell sorting. (B) Levels of the alkaloids detected in leaves, total protoplasts, sorted common mesophyll protoplasts, and sorted idioblast protoplasts. *n*=3. Ajm., ajmalicine; Serp., serpentine; Cath., catharanthine; Vind., vindoline; Anhy., anhydrovinblastine; Vinbl., vinblastine. (C) Experimental design used to generate RNA-seq data for transcriptome assembly. Samples/replicates from the idioblast experimental setup displayed in (A) were used for Illumina sequencing. Samples/replicates from leaf pairs 1 and 4 (counting from the shoot apex) of an experiment including one group of control plants grown in a growth chamber (indoor plants) and another group of plants grown outdoors whilst submitted to natural light/temperature conditions (outdoor plants) were used for Illumina sequencing. Samples/replicates from leaf pairs 2 and 3 of the latter experiment were used for Nanopore sequencing. Hexagons represent leaf samples and circles represent protoplast samples. (D) Summary plots of differentially expressed genes in idioblast protoplasts compared with protoplasts of common mesophyll cells. Left: MA plot showing idioblast up-regulated genes in blue, idioblast down-regulated genes in red-orange, and non-differentially expressed genes in grey. Right: bar plot showing the numbers of differentially expressed genes. Genes with absolute log_2_ FC >1 and adjusted *P*-values <0.05 were considered as differentially expressed. DAT, deacetylvindoline-4-*O*-acetyltransferase; D4H, desacetoxyvindoline-4-hydroxylase; SGD, strictosidine-β-D-glucosidase; STR, strictosidine synthase; SLS, secologanin synthase.

To establish the precise roles of *C. roseus* leaf idioblasts in the MIA pathway, a highly enriched population of leaf idioblast protoplasts was generated by FACS (Fig. 2A), together with a pure population of common mesophyll cell protoplasts. The protoplasts of idioblasts and common mesophyll cells obtained with the applied methodology have been previously shown to retain their *in planta* distinctive identities (Carqueijeiro *et al.*, 2016), and may therefore be used to investigate the precise metabolic and transcriptomic profiles of these two leaf cell types. Idioblast populations contained ~90% of cells showing the intense blue/green autofluorescence and high refraction of the vacuolar content characteristic of *C. roseus* leaf idioblasts, while mesophyll cell populations were constituted exclusively by this cell type.

HPLC-MS analysis of the alkaloid profile of leaves and the several protoplast populations is shown in Fig. 2B (details in Supplementary Fig. S3). The analysis revealed the differential presence in idioblasts of extremely high levels of serpentine, catharanthine, vindoline, and anhydrovinblastine, >100-fold in comparison with common mesophyll cells—from 183-fold for vindoline to 196-fold for catharanthine (Supplementary Table S6). These results are in line with the previously observed staining of idioblasts by alkaloid indicators, and recent alkaloid-specific localization using imaging MS and single-cell metabolomics (Yoder and Mahlberg, 1976; Carqueijeiro *et al.*, 2016; Yamamoto *et al.*, 2019; Li *et al.*, 2023). Vinblastine was detected in idioblasts in low levels but was not detectable in common mesophyll cells. Ajmalicine was detected in leaves and was absent from all protoplast samples. Li *et al.* (2023) also did not detect the presence of ajmalicine in any *C. roseus* leaf protoplast cell population.

The relative proportions of the different alkaloids in idioblast protoplasts were very similar to those observed in leaves and total protoplasts, suggesting that the alkaloid content of idioblasts makes a decisive contribution to the total alkaloid content of leaves (together with laticifers, likely accumulating a similar alkaloid profile, given the similar autofluorescence profile and reactivity with alkaloid reagents; Carqueijeiro *et al.*, 2016). Furthermore, the catharanthine/vindoline ratio is similar in leaves and in idioblasts (1.67 and 1.99, respectively, Supplementary Table S6), clearly indicating that all catharanthine detected in leaves is co-localized with vindoline in idioblasts and likely in laticifers. The presence of high levels of anhydrovinblastine indicates the occurrence of intense dimerization, further confirming co-localization of catharanthine and vindoline. The molar concentration estimated for catharanthine, vindoline, and anhydrovinblastine in idioblasts was in the milimolar range (158, 79, and 32 mM, respectively; Supplementary Table S6), as observed very recently for catharanthine and vindoline by Li *et al.* (2023) using single-cell metabolomics.

### A novel transcriptome assembly refines *C. roseus* gene models

The observed importance of idioblasts for alkaloid accumulation in *C. roseus* leaves (Fig. 2B) prompted the investigation of the differential transcriptomic profile of this cell type, aiming to identify candidate genes for key biosynthetic, transport, and transcriptional regulatory events of the MIA pathway, as well as to build up a global picture of the biology of this cell type. Idioblasts are under-represented within leaf tissues and, therefore, their transcripts may not have been detected in previous *C. roseus* transcriptomic projects, which were the main sources for the identification of coding sequences in the *C. roseus* genomes v1 and v2 (Kellner *et al.*, 2015; Franke *et al.*, 2019). As such, a *de novo* genome-guided transcriptome assembly was performed, using RNA-seq short-read data obtained from the same samples as those analysed for alkaloid levels, complemented with RNA-seq data generated from a second experiment (Fig. 2C). In the latter, a group of control plants was grown in a growth chamber (indoor plants) while another group was grown outdoors, submitted to natural light/temperature conditions (outdoor plants). The RNA-seq experiment design illustrated in Fig. 2C generated a short-read dataset containing 432 million paired-end reads (2 × 125 bp) across 23 biological samples, and a long-read dataset containing 12.6 million reads across 12 biological samples with a median length of 479 bp and an N50 of 731 bp. Consistency in the alignment ratio was observed across all samples, with >90% of the short-read data and 84% of the Nanopore data mapping to the genome v2. An overview of the data can be found in Supplementary Table S7.

The approach used for the genome-guided transcriptome assembly is summarized in Supplementary Fig. S4. After the initial assembly and analysis, several erroneous gene models were detected not only on the individual Stringtie runs but also on the original reference annotation (Supplementary Fig. S5). These artefacts included transcript fusion, fragmented transcripts, and incorrect exon–intron chains. To minimize this problem, Mikado was employed to merge the independent sample assemblies and refine the resulting transcripts (Venturini *et al.*, 2018). This approach corrected several gene models, including models present in the genome v2 reference annotation. The novel assembly contained 59 496 transcriptomic isoforms across 38 788 genes, of which 35 722 were predicted to have an ORF (Supplementary Table S8). This novel transcriptome assembly is hereafter named IDIO+.

Assessment with BUSCO detected 96% of the eudicot core orthologues as complete and 1.3% as fragmented in IDIO+, which represents a significant improvement over the annotation of the reference genome v2, whose scores are 90.6% complete and 3.5% fragmented (Supplementary Table S8) (Franke *et al.*, 2019; Seppey *et al.*, 2019). The IDIO+ BUSCO scores are similar to the CDF97 consensus transcriptome reported by Dugé de Bernonville *et al.* (2015b). However, the high number of gene clusters in CDF97, as well as its high number of duplicated orthologues in BUSCO, indicate redundancy, which was avoided with the genome-guided method used here.

Protein-coding genes were annotated with panzer2 and resulted in GO terms being assigned to 34, 35.1, and 35.5% of the genes for Biological Process, Molecular Function, and Cellular Component, respectively (Toronen *et al.*, 2018). Functional domains and protein families were also annotated using Interpro (Blum *et al.*, 2021) and resulted in 59.9% of the genes being annotated in at least one of the member databases. The overall annotation rate across both methods was 61%. Since the MIA pathway is central to this study, annotation of this pathway’s enzymes, transporters, and transcription factors was performed manually in IDIO+ (Supplementary Table S9).

IDIO+ generated 1128 novel gene loci in comparison with the *C .roseus* genome v2 reference annotation (Supplementary Table S1). Most significantly, 567 of these new genes are expressed in idioblasts, 208 with a log_2_ FC ≥1, increasing the likelihood of discovery of undisclosed transcripts involved in the MIA pathway (Supplementary Table S1; Supplementary Fig. S6). IDIO+ further allowed to uncover alternative splicing events and transcriptomic isoforms, adding new layers of information to the genome.

### Differential gene expression reveals a clear specialization of idioblasts

The improved *C. roseus* reference gene models of IDIO+ were used to perform the characterization of the differential transcriptomic landscape of idioblasts. PCA and hierarchical clustering based on gene expression profiles revealed that idioblasts and common mesophyll cells have clearly distinct transcriptomic profiles (Supplementary Figs S7, S8). This allowed the implementation of DGE analysis of idioblast protoplasts, using the mesophyll protoplast samples as a reference. Robustness of the DGE approach was further guaranteed using an effect size filter (absolute log_2_ FC ≥1) in addition to the significance filter, and by the inclusion in the statistical model of RNA-seq short-read data from the three samples of total protoplasts and from 12 samples of the indoor/outdoor experiment.

**Fig. 3.**
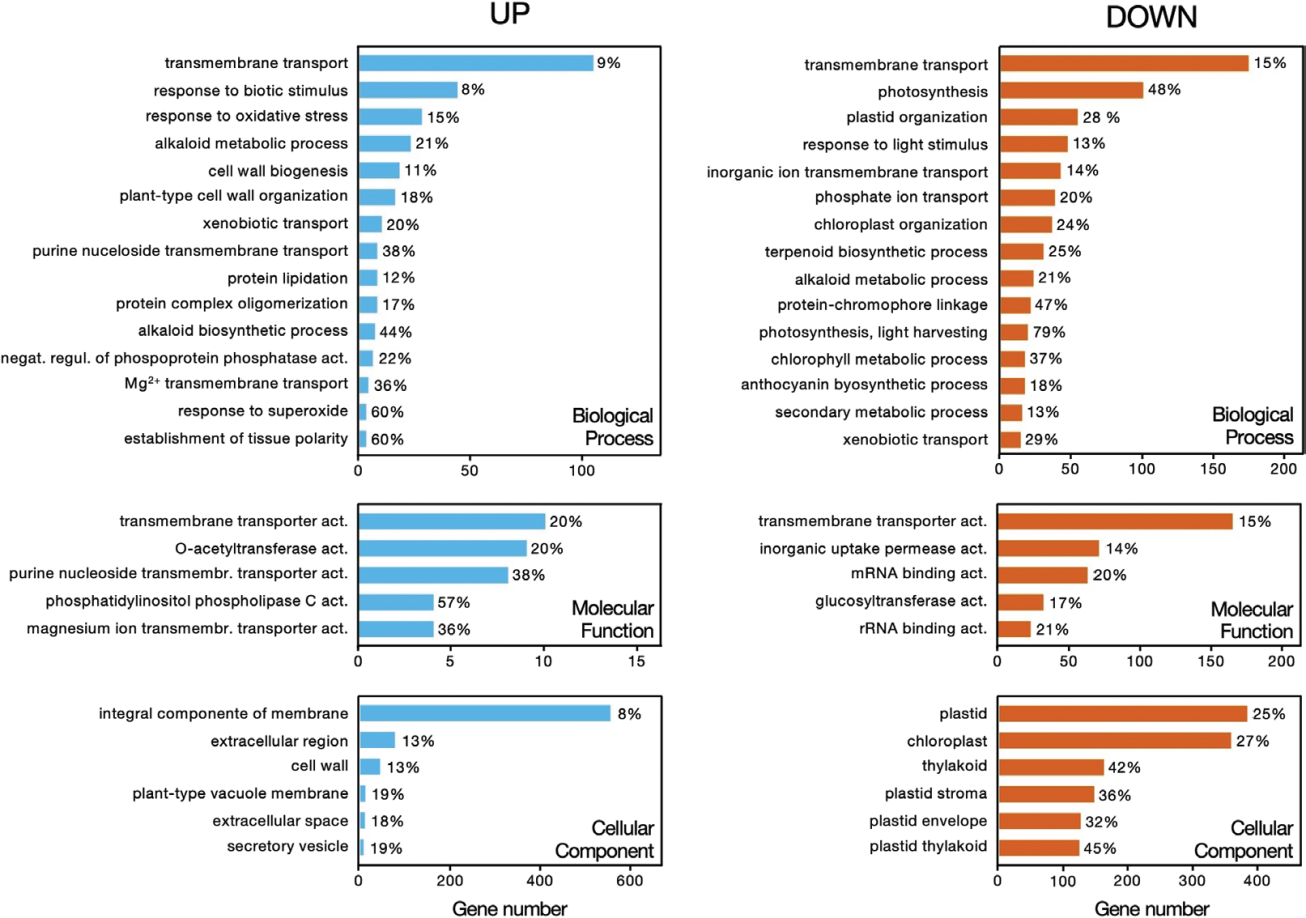
Enrichment analysis of GO terms associated with genes up-regulated (left, blue) and down-regulated (right, red-orange) in idioblast protoplasts compared with common mesophyll cell protoplasts. The percentage of up- or down-regulated genes from each GO term, in relation to the background annotation (list of genes expressed in either idioblasts or common mesophyll protoplasts), is represented next to the respective bar. When a category included >15 significantly enriched terms, only the top 15 (ordered by the number of genes up- or down-regulated) were included.

The idioblastome included 1489 genes overexpressed in this cell type, relative to common mesophyll cells, while 1776 genes were underexpressed (Fig. 2D). The full list of differentially expressed genes can be found in Supplementary Tables S2–S4. The top 25 idioblast up-regulated genes ranked by log_2_ FC and the top 25 genes ranked by expression levels in idioblasts are presented in Tables 1 and 2, respectively. To further understand the underlying roles played by idioblasts, a GO term enrichment analysis was performed for both up- and down-regulated genes (Fig. 3).

**Table 1.** Top 25 up-regulated genes in idioblast protoplasts compared with protoplasts of common mesophyll cells, ranked by log_2_ FC

Gene ID	Log_2_FC	Annotation	TPM
CATHA_25538	12.92	Glycosyltransferase	109.9
CATHA_32840	11.73	Aromatic amino acid decarboxylase 2	53.4
CATHA_24235	11.28	Small GTPase superfamily	109.1
CATHA_18760	10.92	Abscisic stress-ripening protein 2	45.3
CATHA_15400	10.90	**2-Oxoglutarate (2OG) and Fe (II)-dependent oxygenase superfamily protein**	43.9
CATHA_18104	10.77	Beta-amylase	841.4
CATHA_5278	10.23	Sugar transporter	18.4
CATHA_18617	10.23	**Deacetylvindoline-4-*O*-acetyltransferase (DAT)**	65.1
CATHA_12642	10.09	**Tetrahydroalstonine synthase (THAS)**	20.4
CATHA_33484	10.01	DUF679 domain membrane protein 2	695.5
CATHA_10489	10.00	**Serpentine synthase**	75.4
CATHA_38357	10.00	Hypothetical protein	293.2
CATHA_33482	9.95	DUF679 domain membrane protein 2	3725.2
CATHA_30029	9.73	Nodulin MtN21/EamA-like transporter family protein (fragment)	55.1
CATHA_26821	9.73	**Nitrate transporter (NPF2)**	989.6
CATHA_29024	9.71	Premnaspirodiene oxygenase-like (71D)	12.7
CATHA_20042	9.68	Transcription repressor MYB4	612.8
CATHA_33483	9.64	DUF679 domain membrane protein 2	187.1
CATHA_32017	9.60	Major latex protein domain-containing protein	1547.4
CATHA_5494	9.46	Major latex protein	580.6
CATHA_22670	9.46	Phytocyanin domain-containing protein	214.6
CATHA_21679	9.44	**MATE efflux family protein**	258.9
CATHA_24905	9.42	**WRKY transcription factor**	145.3
CATHA_27906	9.38	Purine permease (PUP)	334.7
CATHA_27908	9.29	Purine permease (PUP)	710.1

Manually annotated genes from the MIA pathway are in bold, as are gene families implicated in the MIA pathway. Transcripts per million (TPM) are the average of three idioblast protoplast samples.

**Table 2.** Top 25 up-regulated genes in idioblast protoplasts, compared with protoplasts of common mesophyll cells, ranked by expression levels

Gene ID	Log_2_FC	Annotation	TPM
CATHA_15668	5.86	Major latex protein domain-containing protein	7199.5
CATHA_22715	1.18	1-Aminocyclopropane-1-carboxylate oxidase	5773.2
CATHA_35751	1.83	Beta-fructofuranosidase, insoluble isoenzyme CWINV1-like	4103.5
CATHA_10184	1.82	Hypothetical protein	4078.3
CATHA_33482	9.95	DUF679 domain membrane protein 2	3725.2
CATHA_19823	3.39	Dehydrin	3393.2
CATHA_16221	2.66	Glutamic acid-rich protein-like isoform X1	3347.6
CATHA_13610	1.78	Dehydrin	2585.9
CATHA_7984	2.48	Hexosyltransferase	2382.5
CATHA_9897	1.97	Response to dessication 2	2107.2
CATHA_15979	1.21	J domain-containing protein	2074.7
CATHA_36137	1.21	GDSL-like lipase/acylhydrolase superfamily protein	1973
CATHA_23202	2.88	Histone deacetylase HDT1-like	1964
CATHA_9026	1.09	Hypothetical protein	1786.8
CATHA_18758	2.62	Similar to *Saccharomyces cerevisiae* YMR173W DDR48 DNA damage-responsive protein	1770.5
CATHA_32017	9.60	Major latex protein domain-containing protein	1547.4
CATHA_25357	4.53	**Desacetoxyvindoline-4-hydroxylase (D4H)**	1541.2
CATHA_8113	2.45	Polygalacturonase inhibitor	1501.8
CATHA_26149	7.39	Patatin	1386.8
CATHA_12222	5.02	Glutamine synthetase	1354.1
CATHA_29872	1.23	Nematode resistance protein-like HSPRO2	1348.8
CATHA_34966	1.17	Malic enzyme	1329.5
CATHA_26934	1.84	NAC domain-containing protein	1300.1
CATHA_18632	1.44	Kunitz trypsin inhibitor 2-like	1241.9
CATHA_7086	5.69	Isoflavone reductase homologue	1233.8

Manually annotated genes from the MIA pathway are in bold. TPMs are the average of three idioblast protoplast samples.

The top up-regulated/highly expressed idioblast genes include several genes involved in the biosynthesis of MIAs, namely the idioblast marker genes deacetylvindoline-4-*O*-acetyltransferase (*DAT*) and desacetoxyvindoline-4-hydroxylase (*D4H*), confirming the identity of the idioblast protoplast population and the robustness of the DGE approach. Quantitative PCR (qPCR) confirmation of *DAT* and *D4H* differential expression in a similar FACS idioblast protoplast population has been reported in Carqueijeiro *et al.* (2016). GO enrichment analysis shows that the Biological Process terms ‘alkaloid metabolic process’ (23 genes, 21%) and ‘alkaloid biosynthetic process’ (7 genes, 44%) appear over-represented in idioblasts, while ‘alkaloid metabolic process’ (23 genes, 21%) is also enriched in the down-regulated genes. This fits with the known partition of the *C. roseus* alkaloid pathway between different cell types.

The top idioblast up-regulated genes further include several transporters from gene families involved in alkaloid transport discussed below as MIA candidates [multidrug and toxic compound extrusion (MATE), purine permease (PUP), and nitrate and peptide transporter family (NPF)], and a bidirectional amino acid transporter (MtN21/EamA-like transporter) that may be important to feed idioblasts with amino acid precursors (Table 1). MtN21/EamA-like transporters have been implicated in glutamine transport (Denancé *et al.*, 2014) and their strong up-regulation in idioblasts (~10 log_2_ FC, Table 1) may be related to the fact that glutamine synthetase is also up-regulated (~5 log_2_ FC, Table 1), and is among the top 25 genes with higher expression levels in idioblasts (Table 2). The top enriched term for the GO category Biological Process is ‘transmembrane transport’, for both up- and down-regulated genes, indicating an important differential specialization of idioblasts. Up-regulated ‘transmembrane transport’ represents the term with the highest number of annotated genes (105), with up-regulated genes further including enrichment in ‘xenobiotic transport’, ‘purine nucleoside transport’, and ‘copper ion transmembrane transport’. Transporter activities are also the bulk of GO terms enriched in idioblasts for the category Molecular Function, with ‘integral component of membrane’ being the top enriched term for the category Cellular Component, further indicating a strong distinctiveness of idioblast membranes.

Idioblast vacuoles, a logical final alkaloid accumulation target, also seem to be distinct from surrounding cells, as indicated by the enrichment of the GO term ‘plant-type vacuole’ in up-regulated genes. Moreover, DUF679 domain membrane protein genes, which appear among the genes with the highest log2 fold up-regulation and expression levels in idioblasts (Tables 1, 2), have been implicated in tonoplast and endoplasmic reticulum membrane remodelling, namely during vacuole biogenesis (Kasaras *et al.*, 2012).

Second to transmembrane transport, ‘response to biotic stimulus’ and ‘response to oxidative stress’ are the GO terms more represented in idioblast up-regulated genes (Fig. 3). Accordingly, some of the genes with the highest expression/up-regulation in idioblasts are related to plant defence responses, including several major latex proteins (MLPs), patatin, nematode resistance protein-like, isoflavone reductase, etc. (Tables 1, 2) (Lytle *et al.*, 2009; Cheng *et al.*, 2015; Bártová *et al.*, 2019). Notably, isoflavone reductases are found only in plants and they are considered to be very important in defence against several biotic stresses (Cheng *et al.*, 2015). Several of the top idioblast up-regulated genes have also been implicated in abiotic stress, such as abcisic acid ripening 1 (*ASR1*), dehydrin, and phytocyanin (Golan *et al.*, 2014; Cao *et al.*, 2015).

The idioblast genetic programme seems to be particularly fitted not only to respond to stress, but also to sense stress. The signalling differential specialization of idioblasts is suggested by the high number/‘category %’ of idioblast up-regulated genes annotated with the GO terms ‘integral component of membrane’ (557 genes) and ‘phosphatidylinositol phospholipase C activity’ (57% genes of the category) (Fig. 3). The ~11 log_2_ FC idioblast up-regulation of a small GTPase, which is the third gene with higher log_2_ FC (Table 1), also indicates that idioblasts are quite distinct from mesophyll cells in relation to signalling, since small GTPases are molecular switches that typically function as network nodes integrating broad regulatory inputs into broad effector outputs (Reiner and Lundquist, 2018). A further indication of the higher capacity of idioblasts to sense and respond to stress is the fact that the GO terms ‘cell wall biogenesis’, ‘cell wall thickening’, and ‘cell wall’ are enriched in this cell type, suggesting that these cells respond more efficiently to the protoplasting aggression than common mesophyll cells. Consequently, idioblasts are likely more responsive to cell wall damage resulting from herbivore attack. Another idioblast-enriched term worth mentioning is ‘establishment of tissue polarity’, which potentially includes genes involved in the differentiation of this cell type.

The top enriched GO terms among idioblast down-regulated genes, after ‘transmembrane transport’, include many terms related to photosynthesis, chloroplasts, light, etc., and show an impressive down-regulation of 79% of the genes related to light harvesting (Fig. 3). Furthermore, all enriched GO terms among down-regulated genes for the category Cellular Component are related to plastids/chloroplasts. This indicates that idioblasts are not centrally engaged in light harvesting and carbon fixation, unlike common mesophyll cells. Related to this, the strong idioblast up-regulation of β-amylase, of a sugar transporter, and of β-fructofuranosidase/invertase (Tables 1, 2), responsible for sucrose hydrolysis, further suggest that idioblasts are carbon sinks rather than carbon sources.

### Gene co-expression modules show correlation with alkaloid traits

**Fig. 4.**
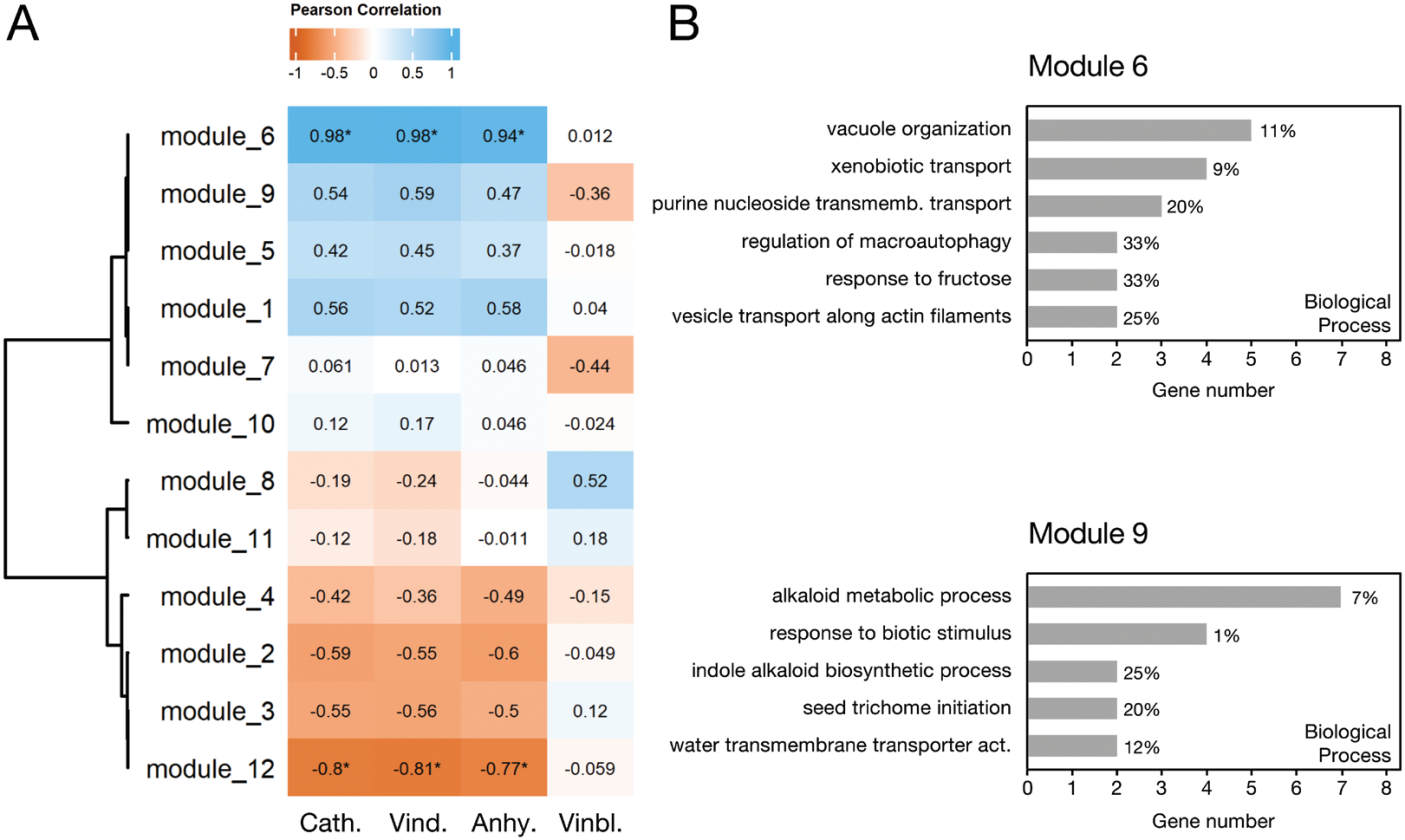
(A) Module–trait correlation analysis of gene co-expression modules with the levels of the alkaloids catharanthine (Cath.), vindoline (Vind.), anhydrovinblastine (Anhy.), and vinblastine (Vinbl.). The clustered heatmap represents correlations between module eigengenes and alkaloid levels, with blue representing positive correlations and red-orange representing negative correlations. Statistically significant correlations are marked with * (adjusted *P*-value <0.05). (B) Enrichment analysis of GO terms (Biological Process) of module 6 and module 9 genes. The percentage of genes from each GO term present in the module, in relation to the background annotation (list of genes included in the co-expression analysis), is represented next to the respective bar. Gene lists per module are given in Supplementary Table S11.

The RNA-seq data of this study originated from two experimental setups (Fig. 2A, C) for which alkaloid levels were also determined. Therefore, the extended RNA-seq dataset of this study was used to generate co-expression modules using weighted gene co-expression network analysis, followed by module–trait analysis inputting the levels of the alkaloids vindoline, catharanthine, anhydrovinblastine, and vinblastine (Supplementary Table S10). Ajmalicine and serpentine levels were not included in the analysis since their biosynthetic pathway is solved, in contrast to the biosynthesis of vinblastine from its precursors anhydrovinblastine, vindoline, and catharanthine. PCA of gene expression levels of all the different samples showed that leaf data from the two experiments dissociated well from protoplast samples (Supplementary Fig. S1). After network construction, module detection, and clustering, 12 gene modules were obtained, and were then used for module–trait analysis (Fig. 4A; Supplementary Table S11). Overall, the results show that catharanthine, vindoline, and anhydrovinblastine levels present a very similar correlation pattern with the different gene modules, in clear contrast to the vinblastine pattern (Fig. 4A).

Over 70% of the genes in modules 6 and 9 are up-regulated in idioblasts (Supplementary Table S11), implying that they might be related to processes specific to these specialized cells. Module 6 presents a significant positive correlation with catharanthine (0.98), vindoline (0.98), and anhydrovinblastine (0.94) traits, suggesting a key role in the regulation of MIA levels (Fig. 4A). Despite the high correlation, dihydroprecondylocarpine synthase (*DPAS*) was the only MIA biosynthetic gene found in this module. Module 6 has an over-representation of genes related to ‘xenobiotic transport’ and ‘purine nucleoside transport’ (Fig. 4B), corresponding, respectively, to MATE and PUP family transporters, which have been implicated in alkaloid transmembrane transport and may be determinants of MIA metabolic flux. Module 9 is closely correlated with module 6 and includes three vindoline pathway genes [tabersonine 16-*O*-methyltransferase (*16OMT*) *DAT*, and *D4H*]. However, the module–trait analysis revealed that the correlation between module 9 and alkaloid levels is not statistically significant (Fig. 4A). GO term analysis revealed that module 9 is enriched in genes involved in alkaloid biosynthesis and response to biotic stimulus (Fig. 4B).

### Differential gene expression confirms specialization of idioblasts in the late MIA pathway

**Fig. 5.**
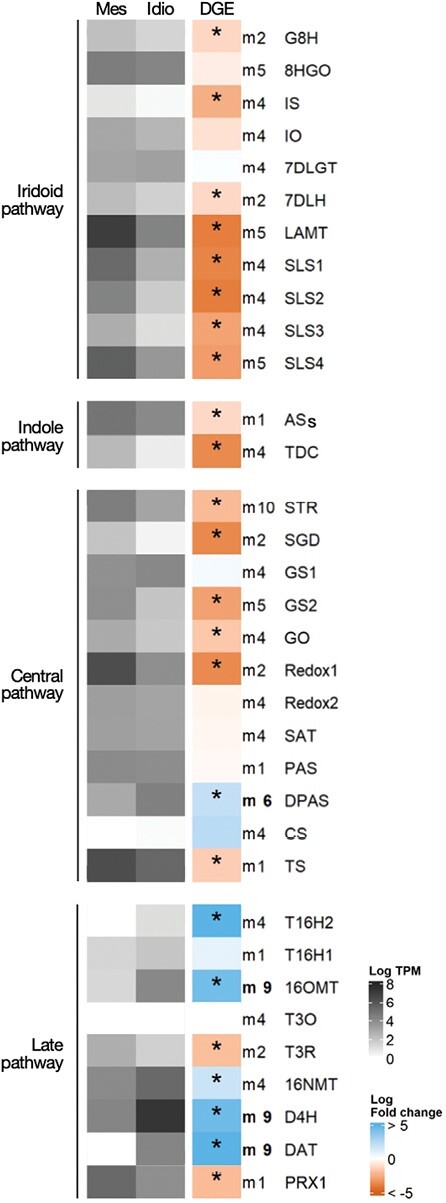
Expression analysis of genes involved in the biosynthesis of the anticancer drug vinblastine. Heatmap grey colours represent the mean log_2_ TPM of three independent biological samples of common mesophyll protoplasts (Mes) and idioblast protoplasts (Idio). DGE (differential gene expression) represents the log_2_ FC for each gene, with blue and red-orange representing, respectively, overexpression and underexpression in idioblast protoplasts compared with common mesophyll protoplasts. Significant differential expression is marked with *. The iridoid pathway: *G8H*, geraniol 8-hydroxylase; *8HGO*, 8-hydroxygeraniol oxidoreductase; *IS*, iridoid synthase; *IO*, iridoid oxidase; *7DLGT*, 7-deoxyloganetic acid glucosyltransferase; *7DLH*, 7-deoxyloganic acid hydroxylase; *LAMT*, loganic acid *O*-methyltransferase; *SLS*, secologanin synthase. The indole pathway: *ASs*, anthranilate synthase; *TDC*, tryptophan decarboxylase. The central pathway: *STR*, strictosidine synthase; *SGD*, strictosidine-β-D-glucosidase; *GS*, geissoschizine synthase; *GO*, geissoschizine oxidase; *SAT*, stemmadenine-*O*-acetyltransferase; *PAS*, precondylocarpine acetate synthase; *DPAS*, dihydroprecondylocarpine synthase; *CS*, catharanthine synthase; *TS*, tabersonine synthase. The late pathway: *T16H1*, tabersonine 16-hydroxylase 1; *T16H*, tabersonine 16-hydroxylase 2; *16OMT*, tabersonine 16-*O*-methyltransferase; *T3O*, tabersonine 3-oxygenase; *T3R*, tabersonine 3-reductase; 16*NMT*, 3-hydroxy-16-methoxy-2,3-tabersonine *N*-methyltransferase; *DAT*, deacetylvindoline-4-*O*-acetyltransferase; *D4H*, desacetoxyvindoline-4-hydroxylase; *PRX1*, class III peroxidase 1. Gene IDs are given in Supplementary Table S9; TPM values in Supplementary Dataset S4; and DGE in Supplementary Table S2.

The results obtained for the differential expression in idioblasts of genes involved in the biosynthesis of the anticancer drug vinblastine are shown in Fig. 5. As already shown in Table 1 above, *D4H* and *DAT* (Fig. 1), responsible for the last two steps of vindoline biosynthesis, are strongly up-regulated in the idioblast cell populations generated in this study. Five of the seven enzymatic steps involved in the late vindoline biosynthetic pathway are up-regulated in idioblasts: tabersonine 16-hydroxylase 2 (*T16H2*), 1*6OMT*, 3-hydroxy-16-methoxy-2,3-dihydrotabersonine *N*-methyltransferase (*NMT*), *D4H*, and *DAT*, responsible for the first, second, fifth, sixth, and seventh steps, respectively (Fig. 1). The fourth step, tabersonine 3-reductase (*T3R*), is down-regulated, and no expression levels were detected for the third step, tabersonine 3-oxygenase (*T3O*), in mesophyll or idioblast protoplasts. Class III peroxidase 1 (*PRX1*), thought to be responsible for the dimerization reaction yielding anhydrovinblastine (Costa *et al.*, 2008), is down-regulated in idioblasts. All other parts of the MIA pathway leading to vinblastine are either down-regulated in idioblasts or not differentially expressed, except *DPAS*, which is up-regulated.

Expression analysis of enzyme genes from MIA pathway branches other than the one leading to the anticancer drug vinblastine is shown in Supplementary Fig. S9. Tetrahydroalstonine synthase 1/2 (*THAS1/2*), vitrosamine synthase (*VAS*), and vincadifformine 19-hydroxylase (*V19H*) are significantly more expressed in idioblasts. Interestingly, heteroyohimbine synthase (HYS), the putative entry enzyme towards serpentine, an alkaloid present in high levels in idioblasts, is not differentially expressed in these cells, presenting very low absolute expression levels. This is the opposite of serpentine synthase (*SS*), which is expressed in differential and absolute high levels in idioblasts (Table 1; Supplementary Fig. S9), in accordance with the observed high serpentine levels in those cells (Fig. 2B). It has been shown that silencing *THAS1* in *C. roseus* leaves induces a significant decrease in ajmalicine levels (Stravinides *et al.*, 2016). This fact, together with the idioblast high levels of *THAS1*, *SS*, and serpentine observed here, suggests that THAS1 may be the enzyme acting together with SS to produce serpentine in idioblasts.

The expression analysis of transporters and transcription factors previously implicated in the MIA pathway is also shown in Supplementary Fig. S9. The ABCG transporter CrTPT2, thought to be responsible for the efflux of catharanthine to the leaf surface, seems to be up-regulated in idioblasts (Yu and DeLuca, 2013), but its expression level is very low in both cell types, removing significance to the differential result. No other transporter or transcription factor implicated in the MIA pathway was up-regulated in idioblasts.

### Differential gene expression reveals candidate genes for missing events of the MIA pathway

Alkaloid analysis (Fig. 2B) confirmed idioblasts as a central accumulating site of *C. roseus* leaf alkaloids and the putative site of vinblastine and vincristine biosynthesis. As such, DGE analysis was screened for candidate genes for vinblastine and vincristine biosynthesis, for transport events necessary for MIA accumulation in idioblast cells and their vacuoles, and for transcription factors potentially regulating vinblastine biosynthesis and MIA metabolic flux.

#### Candidate enzymes

**Fig. 6.**
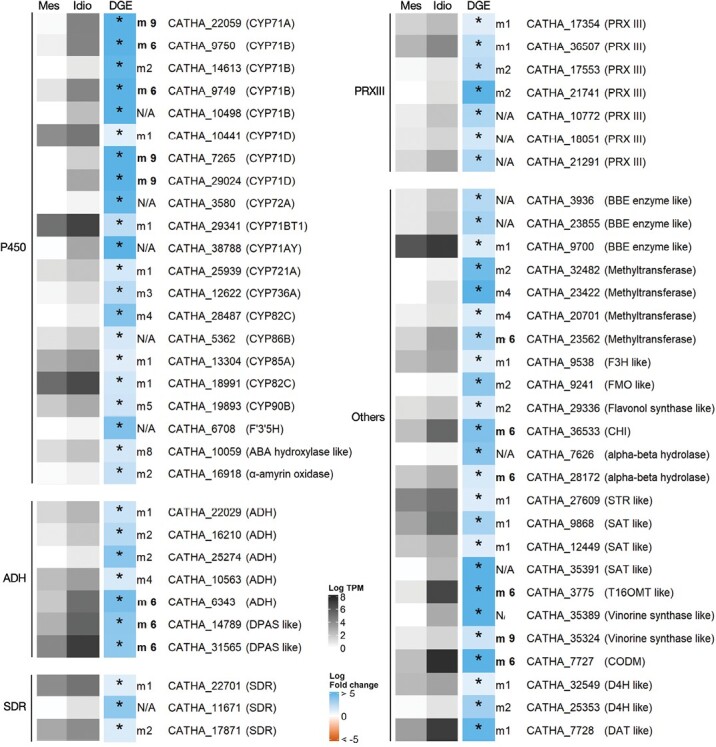
Expression analysis of candidate genes for MIA biosynthetic steps. P450, cytochrome P450s, ADH, alcohol dehydrogenases; SDR, short-chain dehydrogenases; PRX, class III peroxidases. Heatmap grey colours represent the mean log_2_ TPM of three independent biological samples of common mesophyll protoplasts (Mes) and idioblast protoplasts (Idio). DGE (differential gene expression) represents the log_2_ FC for each gene, with blue and red-orange representing, respectively, overexpression and underexpression in idioblast protoplasts compared with common mesophyll protoplasts. Significant differential expression is marked with *. CYP, cytochrome P450; F3ʹ5ʹH, flavonoid 3ʹ5ʹ hydroxylase; ADH, alcohol dehydrogenase; DPAS, dihydroprecondylocarpine synthase; SDR, short-chain dehydrogenases; PRX III, class III peroxidase; BBE, berberine-bridge enzyme; F3H, flavanone 3-hydroxylase; FMO, flavin-containing monooxygenase; CHI, chalcone-flavanone isomerase; STR, strictosidine synthase; SAT, stemmadenine-*O*-acetyltransferase; T16OMT, tabersonine 16-*O*-methyltransferase; CODM, codeine *O*-demethylase like; D4H, desacetoxyvindoline-4-hydroxylase; DAT, deacetylvindoline-4-*O*-acetyltransferase. TPM values are given in Supplementary Dataset S4 and DGE in Supplementary Table S2.

Idioblast up-regulated genes belonging to gene families involved in the biosynthesis of specialized metabolites are shown in Fig. 6. Overall, 22 cytochrome P450s (P450s), seven alcohol dehydrogenases (ADHs), three short-chain dehydrogenases (SDRs), and seven class III peroxidases were identified, together with other enzyme genes such as berberine bridge enzymes, methyltransferases, etc. Several of those genes cluster in module 6 or module 9, further supporting their candidate status for MIA biosynthetic reactions. Two ADHs (CATHA_14789 and CATHA_31565) are similar to *Rauvolfia* spp. vomilenine reductase 2 (81% identity, 98% query coverage, at the amino acid level), which is involved in ajmaline biosynthesis (Geissler *et al.*, 2016). They also present similarity to *C. roseus* DPAS (67% identity 92%, query coverage, at the amino acid level) and they both cluster in module 6 (Fig. 4A).

**Fig. 7.**
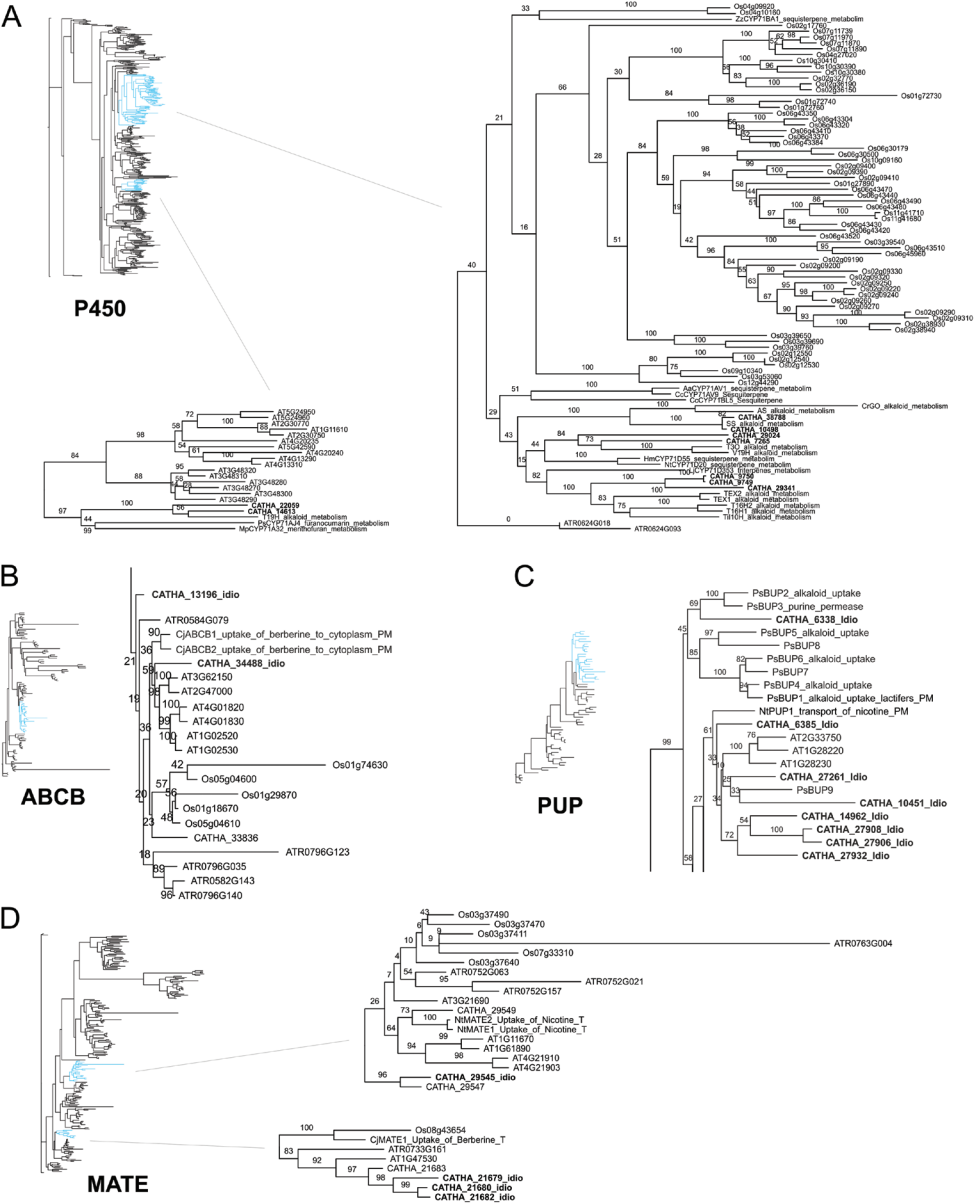
Relevant sections from the phylogenetic trees of selected *C. roseus* candidate proteins for MIA biosynthetic steps and MIA transmembrane transport, together with the whole family proteins from several model species. (A) Cytochromes P450 from clan 71. (B) ABCB transporters. (C) PUP transporters. (D) MATE transporters. MATE proteins previously implicated in alkaloid transport appear in two independent clades. For each gene family tree, the phylogenetic analysis included the selected *C. roseus* proteins up-regulated in idioblasts, the respective family proteins from *Arabidopsis thaliana* (AT), *Oryza sativa* (Os), and *Amborella trichopoda* (ATR) genomes, and the family proteins with known roles in specialized metabolism from other plant origins (Supplementary Tables S12, S14–S16). The full trees can be found in Supplementary Figs S10–S13. CATHA, IDIO+ gene IDs. Species initials: Aa, *Artemisia annua*; Cc, *Cynara cardunculus*; Cj, *Coptis japonica*; Hm, *Hyoscyamus muticus*; Lj, *Lotus japonicus*; Mp, *Mentha piperita*; Nt, *Nicotiana tabacum*; Ps (in C), *Papaver somniferum;* Ps (in A), *Pastinaca sativa*; Sm, *Salvia miltiorrhiza*; Ti, *Tabernanthe iboga*; Zz, *Zingiber zerumbet*. Enzymes (A): AS, alstonine synthase; CYP, cytochrome P450; GO, geissoschizine oxidase; I10H, ibogamine 10-hydroxylase; SS, serpentine synthase; T3O, tabersonine 3-oxygenase; T16H, tabersonine 16-hydroxylase; T19H, tabersonine 19-hydroxylase; TEX, tabersonine epoxidase; V19H, vincadifformine-19-hydroxylase.

In order to further rank the idioblastome P450s, a phylogenetic analysis was performed (Fig. 7A; Supplementary Fig. S10). The P450 family is the largest gene family in plants, with substrate specificity being somewhat phylogenetically conserved (Hamberger and Bak, 2013; Zheng *et al*., 2019). Almost all P450s involved in MIA biosynthesis, as well as the P450 candidates from modules involved in alkaloid metabolism (6 and 9), belong to clan 71. As such, phylogenetic inferences were conducted using only members of this subdivision, to which were added genes from other plant origins with known roles in specialized metabolism (Supplementary Table S12). To generate a robust phylogeny, analysis incorporated the complement of clan 71 P450s from the eudicot and monocot model species *A. thaliana* (AT) and *O. sativa* (Os), and from the angiosperm basal lineage species *A. trichopoda* (ATR). Most of the idioblast P450 candidates are close to other proteins involved in specialized metabolism (Fig. 7A): CATHA_10498 and CATHA_38788 are close to alstonine synthase (AS) and *C. roseus* geissoschizine oxidase (GO); CATHA_29024 and CATHA_7265 are close to vincadifformine 19-hydroxylase (V19H) and T3O; CATHA_9749, CATHA_9750, and CATHA_29341 are close to tabersonine epoxidase 1/2 (TEX1/2) and tabersonine 16-hydroxylase 1/2 (T16H1/2); and CATHA_14613 and CATHA_22059 are in the same subclade as tabersonine 19-hydroxylase (T19H). Additionally, several of these idioblast P450 genes cluster in module 6 or module 9 (Fig. 4A), reinforcing a potential role in the MIA pathway.

#### Candidate transcription factors

**Fig. 8.**
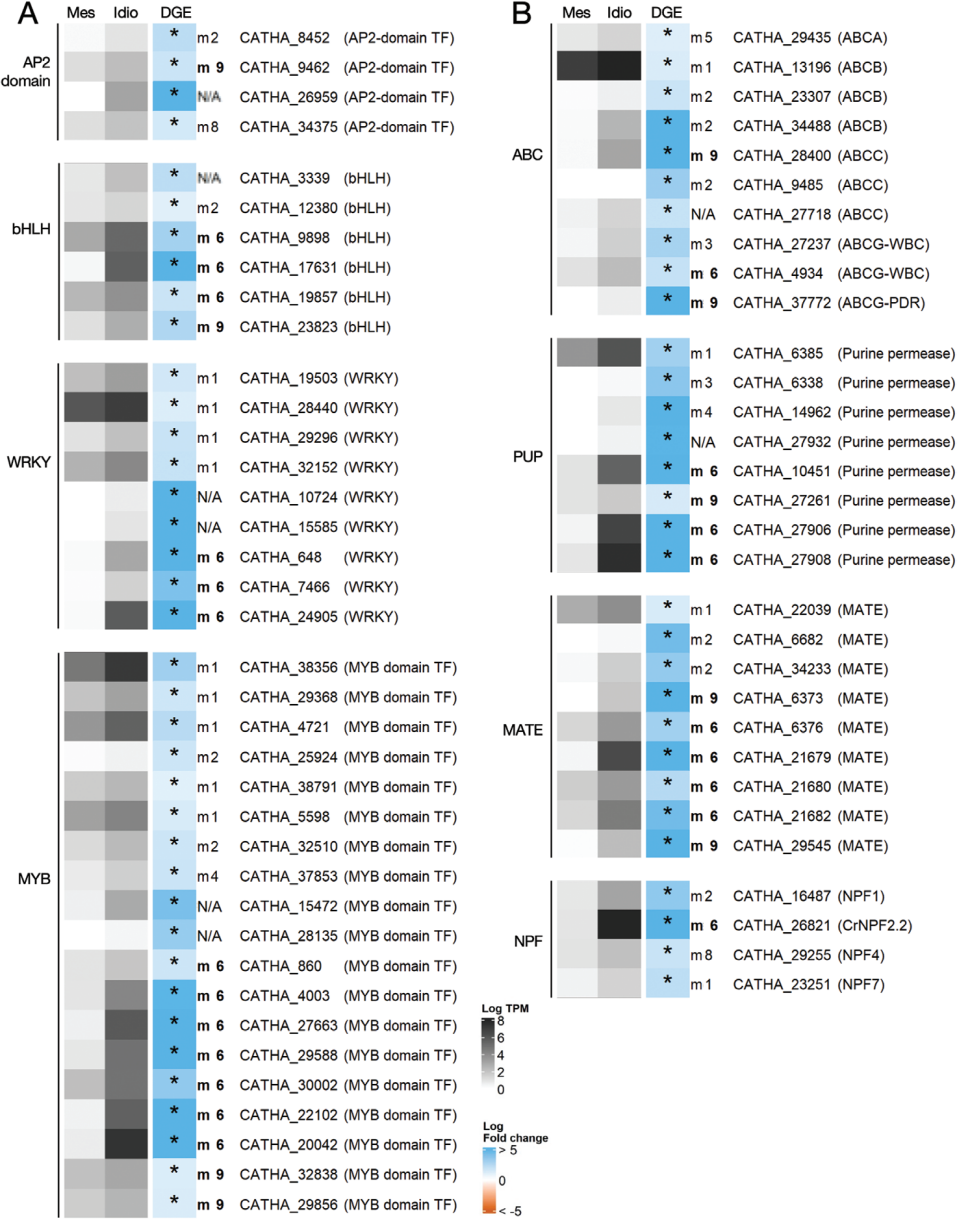
(A) Expression analysis of candidate genes for transcriptional regulation of the MIA pathway. AP2, Apetala 2 domain transcription factors; bHLH, basic helix–loop–helix transcription factors; WRKY, WRKY transcription factors; MYB domain TF, myeloblastosis domain transcription factors. (B) Expression analysis of candidate genes for MIA transmembrane transport events. ABC, ATP-binding cassette transporter; PUP, purine permease; MATE, multidrug and toxic compound extrusion transporter; NPF, nitrate/peptide transporter. Heatmap grey colours represent the mean log_2_ TPM of three independent biological samples of common mesophyll protoplasts (Mes) and idioblast protoplasts (Idio). DGE, differential gene expression, represents the log_2_ FC for each gene, with blue and red-orange representing, respectively, overexpression and underexpression in idioblast protoplasts compared with mesophyll protoplasts. Significant differential expression is marked with *. TPM values are given in Supplementary Dataset S4 and DGE in Supplementary Table S2.

Transcription factors belonging to gene families including members already implicated in the regulation of MIA metabolism in *C. roseus* were searched among up-regulated genes in idioblasts. In total, four AP2-domain (apetala 2), six bHLH (basic helix–loop–helix), nine WRKY genes, and 19 MYB-domain- (myeloblastosis) containing genes were retrieved (Fig. 8A). The full list of up-regulated transcription factors in idioblasts is shown in Supplementary Table S13 and further includes several homeobox genes and plant-specific families of transcription factors, such as NACs, GARPs, and LOBs. Three MYBs listed among candidate transcription factor genes, *CATHA_22102*, *CATHA_29588*, and *CATHA_30002* (Fig. 8A), are included in module 6, associated with alkaloid levels, and their Biological Process GO term annotation includes ‘cell differentiation’ as the unique term. This suggests a possible role in the regulation of the differentiation status of idioblasts and of their specific features concerning alkaloid metabolism. Several other candidate transcription factors clustered in module 6 or 9, including two other MYBs, three WRKYs, and one bHLH (Fig. 8A), all from transcription factor families previously implicated in the regulation of the MIA pathway. They are therefore interesting candidates for the transcriptional regulation of the late MIA biosynthetic steps and metabolic flux.

#### Candidate transporters

The list of genes up-regulated in idioblasts was queried for members of transporter families known to have a role in transmembrane transport of specialized metabolites (Carqueijeiro *et al.*, 2013; Gani *et al.*, 2020). This search yielded several MIA candidates belonging to the transporter gene families MATE (9), ABC (10), PUP (8), and NPF (4) (Fig. 8B). In order to further rank the candidate status of those genes, a phylogenetic analysis was performed, aligning the idioblast candidate proteins of each transporter family with proteins of the respective families previously implicated in transport of specialized metabolites, in *C. roseus* or other plant species (Fig. 7B–D; Supplementary Figs S11, S12; Supplementary Tables S14–S16). Analyses also incorporated the complement of family proteins from *A. thaliana*, *O. sativa*, and *A. trichopoda*.

ABC transporters have been implicated in the transmembrane transport of specialized metabolites, namely the subfamilies ABCB, ABCC, and ABCG (Gani *et al.*, 2020). Phylogenetic analysis of ABCBs revealed that CATHA_34488 clustered with CjABCB1 and CjABCB2, involved in alkaloid cellular uptake in *Coptis japonica* (Shitan *et al.*, 2003, 2013) (Fig. 7B). *CATHA_34488*, like all other ABCB candidates, did not cluster in module 6 or 9, but its relative proximity with *C. japonica ABC1/2* involved in berberine transport is suggestive of alkaloid transport.

Only one candidate NPF transporter (Fig. 8B; *CATHA_26821*) belongs to subfamily 2, which has been implicated in specialized metabolism and includes CrNPF2.9, responsible for export of strictosidine from the vacuoles (Larsen *et al.*, 2017; Payne *et al.*, 2017). *CATHA_26821* is among the top 25 up-regulated genes in idioblasts ranked by log_2_ FC (Table 2); it has high expression levels in idioblasts and clusters in module 6. BLAST search revealed that CATHA_26821 corresponds to a previously studied *C. roseus* NPF2.2 transporter. NPF2.2 was evaluated with negative results for the transport of MIA monoterpenoid precursors, but was not evaluated in relation to MIAs themselves (Larsen *et al.*, 2017). Therefore, the potential role of NPF2.2 in the idioblast import of MIA intermediates should be investigated.

Members of the PUP transporter family were implicated in alkaloid transport from the apoplast to the cytosol, in *Nicotiana tabacum* and *Papaver somniferum* (Jelesko, 2012; Dastmalchi *et al.*, 2019). All the eight candidate PUP transporters up-regulated in idioblasts (Fig. 8B) align in one strongly supported clade (Fig. 7C) containing the previously characterized alkaloid transporters, thus revealing a common evolutionary origin that may have functional significance. Three of the *C. roseus* PUPs cluster in module 6 of the co-expression network (*CATHA_10451*, *CATHA_27906*, and *CATHA_27908*), while one clusters in module 9 (*CATHA_27932*), further strengthening their potential as candidates for MIA transport.

MATE transporters have been implicated in vacuole uptake of alkaloids in *N. tabacum* and *C. japonica* (Otani *et al.*, 2005; Shoji *et al.*, 2009). In *C. roseus*, it has been shown that catharanthine, vindoline, and anhydrovinblastine are transported from the cytosol to the vacuole by a H^+^-antiport system evocative of MATE transporters (Carqueijeiro *et al.*, 2013). There were nine *CrMATE* genes up-regulated in idioblasts (Fig. 8B), and phylogenetic analysis (Fig. 7D) showed that CATHA_29545 aligns in the clade of NtMATE1/2 and that CATHA_21679, CATHA_21680, and CATHA_21682 align in the clade of CjMATE1. *CATHA_29545* clusters in module 9, which includes *DAT* and *D4H*, while *CATHA_21679*, *CATHA_21680*, and *CATHA_21682* cluster in module 6, significantly correlated with alkaloid levels (Fig. 4A). Furthermore, *CATHA_21679* is among the top up-regulated genes in idioblasts (Table 2). These are therefore very strong candidates for the idioblast vacuolar uptake of the different MIAs, the transport event that is potentially the main determinant of MIA metabolic flux in *C. roseus* leaves.

## Discussion

The term ‘idioblast’ is used to describe individual cells that differ in shape, size, wall structure, and/or content from a surrounding homogeneous tissue, and they may appear in any of the tissues of a plant (Foster, 1956). In *C. roseus* leaves, the only plant organ where the anticancer alkaloids are synthesized and accumulated, idioblasts are sparsely and randomly distributed across palisade and spongy parenchyma, are typically larger than their mesophyll counterparts, and display a distinctive refractile and autofluorescent vacuolar content (Mersey and Cutler, 1986; St-Pierre *et al.*,1999; Carqueijeiro *et al.*, 2016). *Catharanthus roseus* leaf idioblasts have been shown to react specifically with alkaloid staining reagents, to specifically express *DAT* and *D4H* responsible for the last steps of vindoline biosynthesis, and to specifically accumulate this alkaloid, one of the monomeric precursors of the anticancer dimeric vinblastine (Fig. 1) (Yoder and Mahlberg, 1976; St-Pierre *et al.*, 1999; Carqueijeiro *et al.*, 2016; Yamamoto *et al.* 2019; Usaki *et al.*, 2022; Li *et al.*, 2023; Sun *et al*., 2023). Here, the FACS isolation of idioblast protoplasts enabled the generation of a new transcriptome assembly, IDIO+, and revealed the differential transcriptome and alkaloid profiles of this cell type, contributing to the understanding of its biology and roles in alkaloid dynamics.

The importance of the anticancer alkaloids produced by *C. roseus* has driven the generation of multiple transcriptomic resources, which were pivotal for the full disclosure of the biosynthetic pathway of vindoline, catharanthine, and serpentine (Góngora-Castillo *et al.*, 2012; Van Moerkercke *et al.*, 2013; Dugé de Bernonville *et al.*, 2015b; Franke *et al.*, 2019). The novel transcriptome IDIO+ includes transcript information from isolated idioblasts, which are low abundant cells in leaves, guaranteeing representation of otherwise rare transcripts. The dataset used also included information from plants grown outdoors, seldom used in research, further enriching transcript representation. Accordingly, 1128 novel gene loci were identified relative to *C. roseus* genome v2, significantly improving gene model information. These results may also positively impact the three improved *C. roseus* genome versions published very recently, after the preparation of this manuscript (Cuello *et al*., 2022; Li *et al*., 2023; Sun *et al*., 2023).

### The idioblastome reveals a unique cell type associated with stress resistance

The differential transcriptome of *C. roseus* leaf idioblasts generated in this work shows the expression of a clearly distinct genetic programme from their surrounding mesophyll cells. Extensive idioblast down-regulation of genes involved in light harvesting/photosynthesis/chloroplasts, together with the high up-regulation of specific carbon metabolism proteins, strongly suggest that this cell type is a carbon sink, opposite to the surrounding mesophyll cells (Fig. 3; Table 2). Significantly, higher activity of β-fructofuranosidase/invertase, as observed in idioblasts, has been reported for sink organs together with higher expression of specialized metabolism (Sturm, 1999; Nishanth *et al.*, 2018).

GO enrichment analysis indicates that idioblasts show a radically different transport protein landscape (Fig. 3) that certainly is correlated with the distinct behaviour concerning carbon metabolism and the specialization in alkaloid accumulation (Fig. 2B). Likewise, idioblast up-regulation profiles show the presence of gene families implicated in alkaloid transport and of genes indicative of an intense nitrogen (e.g. glutamine) transport and metabolism that may feed idioblasts with an abundance of amino acids, such as the MIA precursor tryptophan (Table 1; Fig. 8B). Interestingly, a high glutamine production in Arabidopsis was previously associated with stress responses (Ji *et al.*, 2019), another highlighted feature of idioblasts.

Up-regulated and highly expressed genes in idioblasts (Tables 1, 2), together with GO term enrichment (Fig 3), clearly indicate a specialization of this cell type in biotic and abiotic stress resistance. In particular, the major presence of three MLPs in idioblasts should be highlighted, with *CATHA_15668* corresponding to the gene with the highest expression in this cell type (Table 2). MLPs are plant-specific proteins that were first identified as two of the most abundant proteins in the alkaloid-accumulating latex of opium poppy (Nessler *et al.*, 1985), and they have been implicated in drought and salt tolerance, as well as in the induction of resistance to pathogens (Fujita and Inui, 2021). Their major presence in the alkaloid-accumulating idioblasts of *C. roseus* is clearly related to a similar presence in the alkaloid-accumulating poppy latex, and suggests a common role, most probably defence against herbivore attacks and subsequent pathogen exposure. The action mechanisms of MLPs and their potential use in crop protection and improvement of stress resilience should be investigated.

Overall, the idioblastome reveals a unique cell type, associated with resistance to abiotic and biotic stresses. The overall functional portrait highlighted by the idioblastome, together with the high levels of alkaloids (Fig. 2B), whose animal toxicity and bitterness guarantee potent herbivore-deterrent activity, indicate that *C. roseus* leaf idioblasts must be instrumental in responses to and resistance against environmental challenges.

### A new paradigm for alkaloid accumulation in *C. roseus* leaves

In this work, analysis of the alkaloid profile of idioblast protoplasts revealed the differential presence of extremely high levels of serpentine, catharanthine, vindoline, and anhydrovinblastine, with relative proportions of the different alkaloids suggesting their major co-accumulation in idioblasts within the leaf (Fig. 2B; Supplementary Table S6). This confinement of specialized metabolism to particular cell types is a recurrent feature in plants, with examples including morphine in poppy laticifers, glucosinolate metabolism in Arabidopsis myrosin cells and S-cells, and terpenoids in glandular trichomes of ~30% of vascular plants (Glas *et al.*, 2012; Beaudoin and Facchini, 2014; Shirakawa and Hara-Nishimura, 2018). Such compartmentation, together with vacuole accumulation of most specialized metabolites, including MIAs, has been considered a strategy to manage the burdens of overproduction of specialized metabolites, including competition for resources, metabolic imbalance, and potential self-intoxication (Carqueijeiro *et al.*, 2013; Chezem and Clay, 2016).

Alkaloid concentrations in *C. roseus* idioblasts were further shown here to reach millimolar levels, similar to the values reported recently by Li *et al.* (2023). These high alkaloid concentrations are likely stored inside the idioblast vacuole, avoiding self-toxicity effects (Carqueijeiro *et al.*, 2013), and should guarantee a high local toxicity leading to high defence efficiency, when an insect herbivore bites the *C. roseus* leaf. This extreme alkaloid defence specialization of idioblasts may be incompatible with normal mesophyll cell metabolism, justifying the differentiation of this cell type and the observed partitioning of the MIA pathway.

Most studies show that *C. roseus* leaves accumulate high levels of catharanthine and vindoline, with high levels of anhydrovinblastine also being frequently reported, as detected in the present study (Balsevich and Bishop, 1989; Naaranlahti *et al.*, 1991; Tang *et al.*. 2009; Pan *et al.*, 2016; and, in our lab, by Sottomayor *et al.*, 1998; Costa *et al.* 2008; and Carqueijeiro *et al.*, 2013). In these cases, the true bottleneck in relation to the accumulation of the anticancer alkaloids is the conversion of anhydrovinblastine into vinblastine, a reaction that has escaped characterization so far. There are other reports where anhydrovinblastine levels are low, undetected, or not mentioned, indicating that the dimerization reaction may also be an important bottleneck for specific *C. roseus* cultivars/growing conditions (e.g. Mersey and Cutler, 1986; Murata and De Luca, 2005; Roepke *et al.*, 2010; Yu and De Luca, 2013; Dugé de Bernonville *et al.*, 2017; Yamamoto *et al.*, 2019; Li *et al.*, 2023). In the plants used here, the high dimerization activity is robustly co-regulated with the biosynthesis of the monomeric precursors, opposite to vinblastine levels (Fig. 4A).

The co-accumulation of catharanthine and vindoline in idioblasts observed in this study challenges previous data suggesting that catharanthine is accumulated at the epidermal cuticle, away from the idioblast-accumulated vindoline (Roepke *et al.*, 2010; Yu and De Luca, 2013). However, the claimed specific accumulation of catharanthine in the leaf surface was based on a leaf chloroform dipping technique, which was later shown to destroy the cell integrity of *C. roseus* leaves, leading to alkaloid cell leakage (Abouzeid *et al.*, 2019). Moreover, previous work by the same research group, using an uncontested abrasion technique to isolate the epidermis/cuticle, had shown that catharanthine was accumulated in the remnant leaf body (Murata and De Luca, 2005). Spatial co-accumulation of catharanthine and vindoline inside idioblasts has actually been reported for a long time based on centrifugation samples enriched in idioblast protoplasts (Mersey and Cutler, 1986), and was very recently confirmed using advanced imaging and single-cell MS technologies (Yamamoto *et al.*, 2019; Li *et al.*, 2023). In agreement with co-accumulation of catharanthine and vindoline, the treatment of *C. roseus* leaves with alkaloid staining reagents has consistently shown reaction with idioblasts and laticifers, but never with the epidermis or the leaf surface (Yoder and Mahlberg, 1976; Carqueijeiro *et al.*, 2016; Usaki *et al.*, 2022). Carqueijeiro *et al.* (2013) showed that mesophyll protoplasts accumulate high levels of vindoline, catharanthine, and anhydrovinblastine, indicating cellular accumulation of catharanthine and co-localization with vindoline to yield anhydrovinblastine. Additionally, it was shown that *C. roseus* mesophyll vacuoles perform specific uptake of all three alkaloids through a H^+^-antiport system, reinforcing the evidence supporting cellular accumulation of catharanthine. Finally, the presence of high levels of anhydrovinblastine in *C. roseus* leaves, indicative of catharanthine and vindoline co-localization, has been repeatedly reported (references above), spanning at least three different cultivars across six distinct geographical locations. The accumulation of catharanthine in idioblasts indicates that it must be transported from the epidermis to idioblasts, since catharanthine synthase (CS) has been recently shown to be preferentially expressed in epidermal cells (Li *et al.*, 2023).

**Fig. 9.**
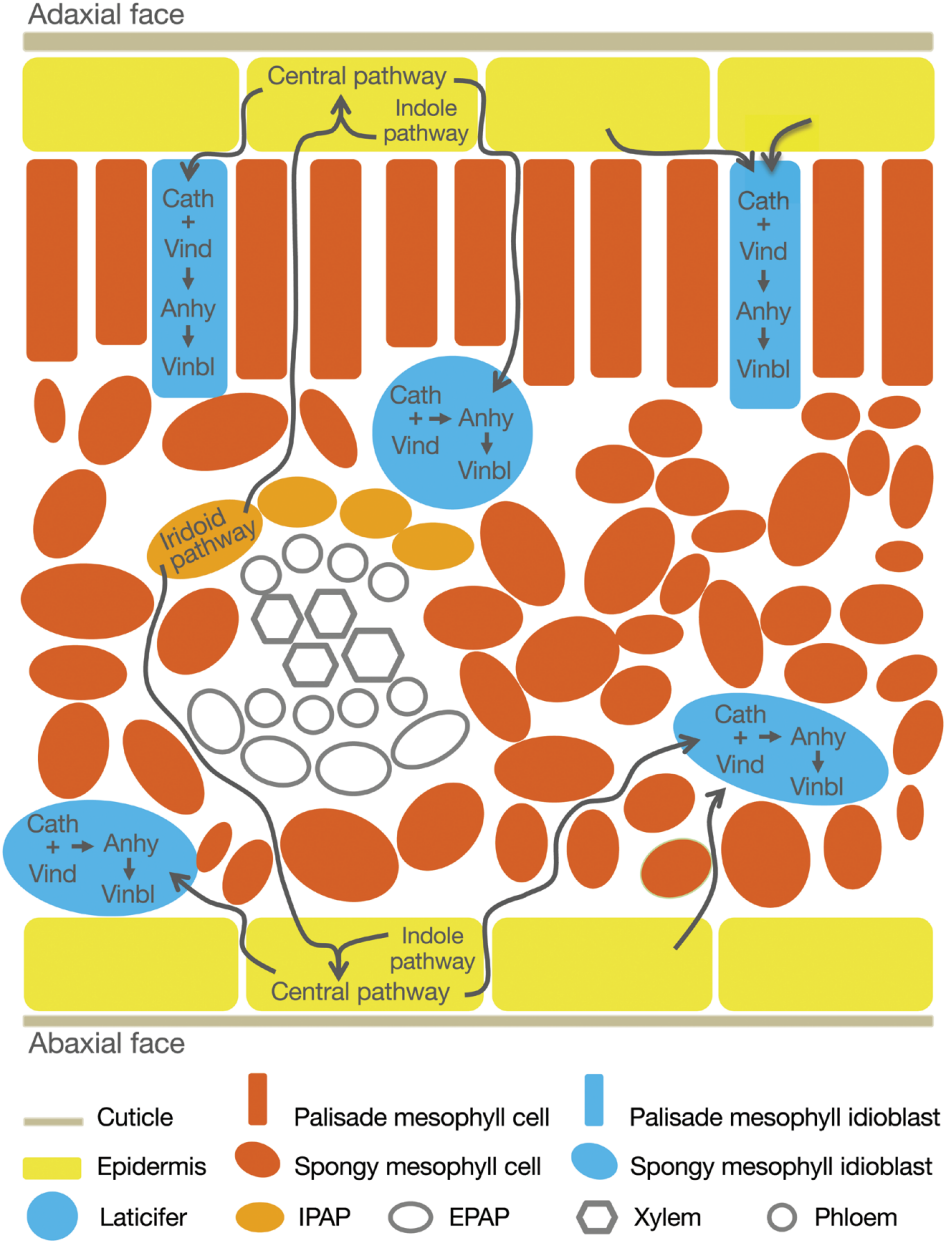
Model proposed for the architecture of the MIA pathway in *C. roseus* leaves. Iridoid precursors are produced in IPAP cells and transported to epidermal cells where the central pathway takes place, also incorporating indole precursors. Catharanthine and a vindoline precursor are transported to mesophyll idioblasts and laticifers, where those monomeric alkaloids accumulate in high levels. The dimerization into anhydrovinblastine and the subsequent conversions to vinblastine and vincristine, the true bottlenecks of the pathway, also take place in idioblasts and laticifers. Anhy, anhydrovinblastine; Cath, catharanthine; EPAP, external phloem-associated parenchyma; IPAP, internal phloem-associated parenchyma; Vinbl, vinblastine; Vind, vindoline. For pathway details, see Fig. 1.

This study, together with abundant reported evidence (notably Yamamoto *et al.*, 2019 and Li *et al.*, 2023), establishes idioblasts as a central accumulation target of alkaloids in *C. roseus* leaves, and rationally the home of the key biosynthetic steps leading to the dimeric anticancer alkaloids, of transporters determining alkaloid metabolic flux, and of instrumental transcriptional regulators of the MIA pathway (Fig. 9). As such, the low levels of the dimeric anticancer alkaloids vinblastine and vincristine should not be credited to the spatial seclusion of their monomeric precursors catharanthine and vindoline, as often stated in review literature (Courdavault *et al.*, 2014; De Luca *et al.*, 2014; Qu *et al.*, 2019; Kulagina *et al.*, 2022), but rather to elusive biosynthetic mechanisms whose final clarification should be searched for in idioblasts. Based on their similar fluorescent properties, reactivity with alkaloid reagents, and *D4H/DAT* expression profile (St-Pierre *et al.*, 1999; Carqueijeiro *et al.*, 2016), laticifers are most likely an alkaloid hot spot akin of idioblasts.

### The idioblastome reveals new insights into the MIA pathway and establishes a roadmap towards its full elucidation

The differential expression of MIA biosynthetic genes in idioblasts shows that the indole, iridoid, and central MIA pathways are down-regulated in this cell type, while most of the late pathway, encompassing the conversion of tabersonine into vindoline, is up-regulated (Figs 1, 5). It is also evident that three of the branching pathways competing with the central pathway for the common strictosidine aglycone substrates are up-regulated in idioblasts (Fig. 1; Supplementary Fig. S9). How the plant regulates the channelling of strictosidine aglycone into the divergent structural classes stemming from this central precursor is precisely one of the main riddles of the MIA pathway. Metabolic channelling resulting from interaction between strictosidine-β-D-glucosidase (SGD) and subsequent enzymes is probably one of the mechanisms involved (Stravinides *et al.*, 2015, 2016). Our results suggest that differential subcellular localization may also be important to determine the fate of strictosidine aglycone, since geissochizine synthase 1 (*GS1*) transcripts, feeding the central pathway towards catharanthine and tabersonine, have been shown to be enriched in epidermal extracts (Tatsis *et al*., 2017; Qu *et al.*, 2018a), while the competing *THAS1/2* and *VAS* on are here shown to have differential higher expression in idioblasts. Strictosidine aglycone isomers are very unstable and toxic, and it has always been assumed that they are promptly converted on-site by subsequent enzymes (Guirimand *et al.*, 2010; Stravinides *et al.*, 2016). The high expression levels of *THAS1/2*, *VAS*, and *SS* in idioblasts, together with the high levels of the alkaloid product serpentine, suggest that the entry enzymes of the MIA central pathway, STR and SGD, although down-regulated in idioblasts, are also operating in this cell type.

An intriguing observation is the up-regulation of *DPAS* in idioblasts, opposite to the immediately previous and subsequent enzymes in the pathway (Figs 1, 5). This enzyme has been proposed to generate the unstable intermediate dihydroprecondylocarpine acetate (Caputi *et al.*, 2018), which also acts as a hub substrate originating at least three different products (Fig. 1). *DPAS* is also the only enzymatic step of the MIA pathway found in co-expression module 6 (Supplementary Table S11), which is the only module showing a significant positive correlation with the levels of the main alkaloids accumulated in *C. roseus* leaves/idioblasts (Fig. 4A). DPAS may therefore be a key step influencing decisively the metabolic flux into the production of catharanthine and vindoline, and deserves further attention.

*PRX1*, encoding a class III peroxidase putatively responsible for the coupling reaction of catharanthine and vindoline to yield anhydrovinblastine, is down-regulated in idioblasts (Fig. 5). *PRX1* is also not co-expressed with *D4H* and *DAT*, in module 9, nor clustered in module 6, which has a high correlation with anhydrovinblastine levels (Fig. 4A). Like most class III peroxidases, evidence suggests that PRX1 is not a specific enzyme and has a wide range of roles, including scavenging of excess hydrogen peroxide generated during photosynthesis at the expense of a range of different substrates (Ferreres *et al.*, 2011). Most photosynthetic processes are greatly down-regulated in idioblasts (Fig. 3), which could explain the lower expression of PRX1 in such cells, independently of its function as the dimerizing enzyme. Otherwise, it is not impossible that although PRX1 is capable of performing the coupling reaction *in vitro*, there is a more specific enzyme performing that reaction *in vivo*, in idioblasts.

A striking outcome of module–trait correlation analysis is the radically different correlation behaviour of vinblastine in comparison with all its immediate precursor alkaloids (Fig. 4A). This indicates that the unknown enzyme responsible for the reaction leading to this valuable anticancer alkaloid might not be co-expressed with the other MIA pathway steps occurring in idioblasts. Moreover, no significant correlation was found between vinblastine and any of the gene co-expression modules, in line with its elusive biosynthesis, whose key ultimately resides in idioblasts. Likewise, the gaps concerning the bottleneck biosynthetic steps leading to the anticancer vinblastine and vincristine should be among the candidate enzymes shown in Fig. 6, featuring two DPAS-like ADHs and several versatile P450s.

There seems to be a significant overlap between the transcriptional regulatory networks governing specialized metabolism and the development of associated cell types (Chezem and Clay, 2016). Our data indicate that *C. roseus* mesophyll idioblasts are specialized in the late steps of the MIA pathway and centralize MIA accumulation in *C. roseus* leaves. Therefore, the transcription regulatory network determining idioblast differentiation and the maintenance of their differentiation status is most likely key for the regulation of the late MIA pathway and MIA metabolic flux. Idioblast up-regulated transcription factors include strong candidates for such important regulatory functions (Fig. 8A).

Correlation analysis of gene co-expression modules with alkaloid traits showed that only module 6 has a significant correlation with alkaloid levels (Fig. 4A). This module includes mostly genes up-regulated in idioblasts, it includes only one MIA biosynthetic gene and it encompasses many transporter genes (Supplementary Table S11). This result indicates the relevance of idioblasts for alkaloid accumulation in *C. roseus* leaves and suggests that alkaloid levels are not determined by the expression levels of biosynthetic enzymes, but rather by the intensity of metabolic flux generated by alkaloid transmembrane transport. As such, the transporter candidates identified in the idioblast complement of genes (Fig. 8B) hold an exceptional potential regarding the understanding and improvement of alkaloid metabolic flux in *C. roseus*. Significantly, module 6 includes several candidates with close phylogenetic relationships with transporters previously shown to be involved in alkaloid transport in other species (Figs 7B–D, 8B, see the Results).

Given the exclusive accumulation of high levels of alkaloids in *C. roseus* leaf idioblasts (together with laticifers) it seems obvious that this cell type acts as a sink of whatever alkaloids are produced elsewhere in the leaves. On the other hand, the idioblast expression profile shows that these cells are particularly active in the last steps of biosynthesis of serpentine and vindoline, and are the specific home of the critical biosynthetic steps leading to the dimeric anticancer alkaloids from the monomeric precursors vindoline and catharanthine. Therefore, the idioblastome holds the key to the identification of the genes encoding the enzymes responsible for the bottleneck biosynthetic steps leading to the anticancer drugs vinblastine and vincristine, the transporters determining alkaloid metabolic flux, and the transcription factors regulating the expression levels of both MIA enzyme and transporter genes. The present work provides a comprehensive roadmap towards the deep understanding of the MIA pathway complexity and the future implementation of successful strategies to increase the levels of the *C. roseus* anticancer alkaloids.

## Supplementary data

The following supplementary data are available at *JXB* online.

Dataset S1. GTF file with the IDIO+ full mRNA gene models (including all alternative splicing isoforms) and the respective localization coordinates in *C. roseus* genome v2. This was the file used for DGE.

Dataset S2. FASTA file including IDIO+ mRNA coding sequences.

Dataset S3. TSV file including the functional annotation of IDIO+.

Dataset S4. CSV file including the TPM expression levels of IDIO+ genes in the samples used for DGE.

Fig. S1. Principal component analysis (PCA) of all the Illumina short-read transcriptomic datasets (Fig. 2C).

Fig. S2. Scale-free topological indices at various soft-thresholding powers for the co-expression network used in the module–trait correlation analysis of Fig. 4A.

Fig. S3. Extracted ion chromatograms of a typical sample of idioblast protoplasts and the extracted ion chromatograms of alkaloid standards used for alkaloid identification.

Fig. S4. Summary of the approach used for genome-guided transcriptome assembly.

Fig. S5. Screenshot obtained with the genome browser Jbrowse (jbrowse.org) depicting an example of an erroneous gene model of a MATE protein present in the reference genome annotation (CRO_T123311).

Fig. S6. Gene expression heat map of the 1128 novel gene loci identified in IDIO+.

Fig. S7. Principal component analysis (PCA) of the transcriptomic datasets generated for the different tissue and cell samples involved in the isolation of idioblasts (Fig. 2C, upper part).

Fig. S8. Hierarchical clustering of the different tissue and cell samples involved in the isolation of idioblasts, based on the regularized log transformation of normalized read counts per gene.

Fig. S9. Expression analysis of genes that have been implicated in MIA branching pathways (Fig. 1), in MIA transmembrane transport, and in transcriptional regulation of the MIA pathway.

Fig. S10. Phylogenetic analysis of cytochrome P450s from clan 71 up-regulated in *C. roseus* idioblasts.

Fig. S11. Phylogenetic analysis of ABCB transmembrane transporters up-regulated in *C. roseus* idioblasts.

Fig. S12. Phylogenetic analysis of PUP transmembrane transporters up-regulated in *C. roseus* idioblasts.

Fig. S13. Phylogenetic analysis of MATE transmembrane transporters up-regulated in *C. roseus* idioblasts.

Table S1. List of novel genes identified by IDIO+.

Table S2. Genes up-regulated in idioblast protoplasts in comparison with protoplasts of common mesophyll cells.

Table S3. Genes down-regulated in idioblast protoplasts in comparison with protoplasts of common mesophyll cells.

Table S4. Genes not significantly differentially expressed in idioblast protoplasts in comparison with protoplasts of common mesophyll cells.

Table S5. Best fitting evolutionary model used for the phylogenetic analyses performed for each gene family.

Table S6. Levels of the alkaloids detected in leaves, total protoplasts, sorted common mesophyll protoplasts, and sorted idioblast protoplasts.

Table S7. Overview of the sequencing data used for transcriptome assembly.

Table S8. Comparison of the IDIO+ transcriptome assembly with the *C. roseus* genome v2 annotation and the consensus CDF97 transcriptome.

Table S9. Manual annotation of the MIA pathway genes in the IDIO+ transcriptome.

Table S10. Levels of alkaloids for the different samples used for weighted gene co-expression network analysis.

Table S11. Gene lists of co-expression modules of Fig. 4.

Table S12. Proteins involved in specialized metabolism used in the phylogenetic analysis of the P450 family.

Table S13. Full list of putative transcription factors up-regulated in idioblasts and candidate for regulation of MIA metabolism.

Table S14. Proteins involved in specialized metabolism used in the phylogenetic analysis of the ABCB family.

Table S15. Proteins involved in specialized metabolism used in the phylogenetic analysis of the PUP family.

Table S16. Proteins involved in specialized metabolism used in the phylogenetic analysis of the MATE family.

erad374_suppl_Supplementary_Figs_S1-S12_Tables_S1-S13

## Data Availability

The RNA-seq data have been deposited in NCBI’s Gene Expression Omnibus (Edgar *et al.*, 2002) and are accessible through GEO Series accession number GSE217852 (https://www.ncbi.nlm.nih.gov/geo/query/acc.cgi?acc=GSE217852). The full IDIO+ transcriptome (TSA, transcriptome shotgun assembly), including coding, non-coding, and splicing variants, has been deposited at DDBJ/EMBL/GenBank under accession number GKIJ00000000. The version described herein is the first version, GKIJ01000000. All other data supporting the findings of this study are available within the paper and within its supplementary data published online.

## Abbreviations

ABC: ATP-binding cassette
anhydrovinblastine: α-3ʹ,4ʹ-anhydrovinblastine
BUSCO: Benchmarking set of Universal Single-Copy Orthologs
D4H: desacetoxyvindoline-4-hydroxylase
DAT: deacetylvindoline-4-*O*-acetyltransferase
DGE: differential gene expression
DPAS: dihydroprecondylocarpine synthase
FACS: fluorescence-activated cell sorting
GO: Gene Ontology
MATE: multidrug and toxic compound extrusion
MIA: monoterpenoid indole alkaloid
NPF: nitrate and peptide transporter family
P450: cytochrome P450
PCA: principal component analysis
PRX1: class III peroxidase 1
PUP: purine permease
RT: retention time
SGD: strictosidine-β-D-glucosidase
SS: serpentine synthase
STR: strictosidine synthase
T3O: tabersonine 3-oxygenase
T3R: tabersonine 3-reductase
THAS: tetrahydroalstonine synthase
TPM: transcript count per million
VAS: vitrosamine synthase.
