# Supplementary material for: The leaf idioblastome of the medicinal plant Catharanthus roseus is associated with stress resistance and alkaloid metabolism
Source: J Exp Bot. 2023 Oct 7;75(1):274–99. doi: 10.1093/jxb/erad374 (PMC10735432; doi:10.1093/jxb/erad374)
Supplement: erad374_suppl_Supplementary_Figs_S1-S12_Tables_S1-S13 [file erad374_suppl_supplementary_figs_s1-s12_tables_s1-s13.zip › Suppl Figs_S1-S12 and Table S1-S13/erad374_suppl_Supplementary_Figures_S1-12.pdf]

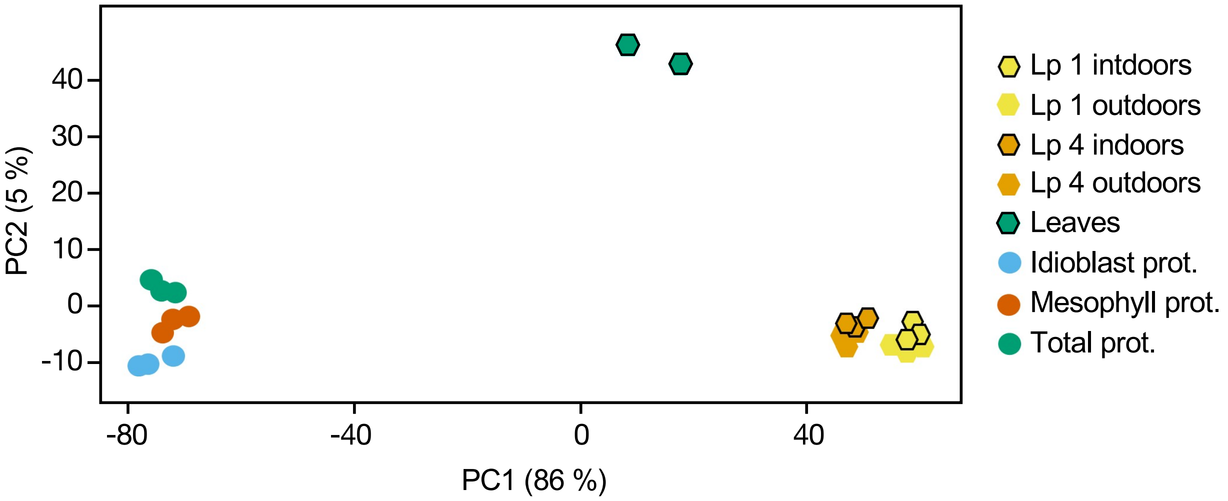

Supplementary Fig. S1. Principal Component Analysis (PCA) of all the Illumina short-read transcriptomic datasets (Fig. 2C). Analysis was based on the regularized log transformation of normalized read counts per gene. Lp 1 and 4 – leaf pair 1 and 4 counting from the shoot apex. Indoors and outdoors – plant leaves from an experiment including one group of control plants grown in a growth chamber (indoor plants) and another group of plants grown outdoors, submitted to natural light and temperature conditions (outdoor plants). Hexagons represent leaf samples and circles represent protoplast samples. Outlined hexagons correspond to indoor plants.

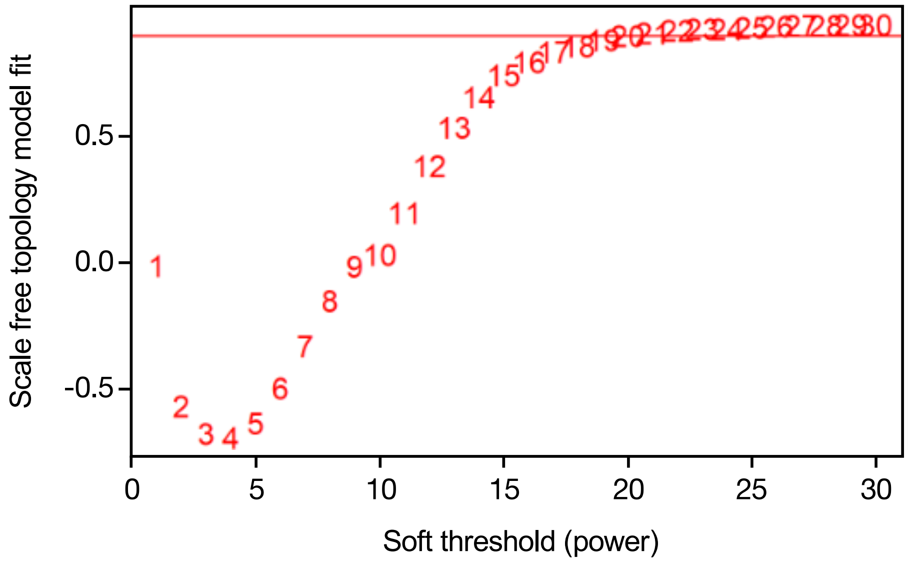

Supplementary Fig. S2. Scale-free topological indices at various soft-thresholding powers for the co-expression network used in the module-trait correlation analysis of Fig. 4A.

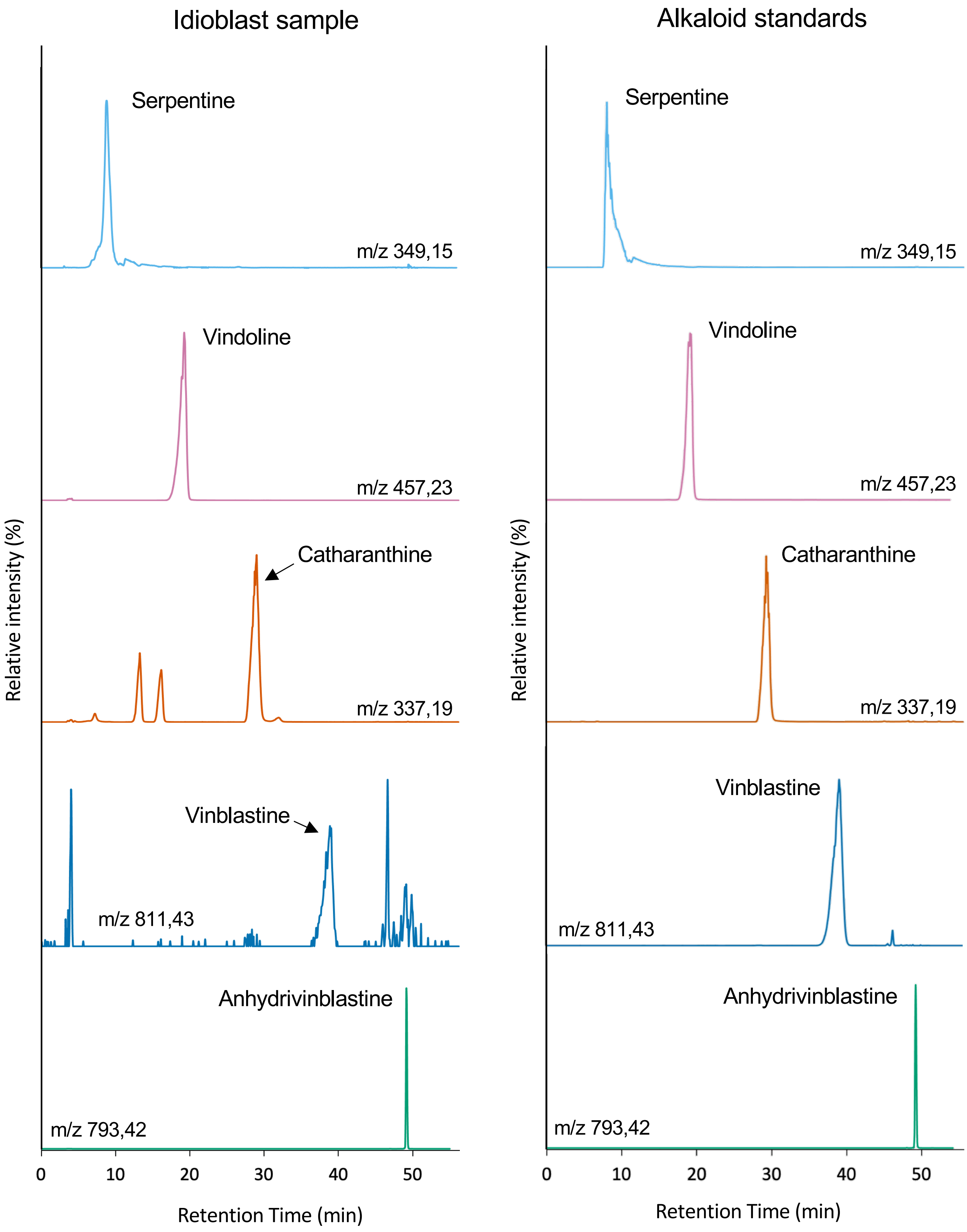

Supplementary Fig. S3. Extracted ion chromatograms of a typical sample of idioblast protoplasts (left) and the extracted ion chromatograms of alkaloid standards used for alkaloid identification (right).

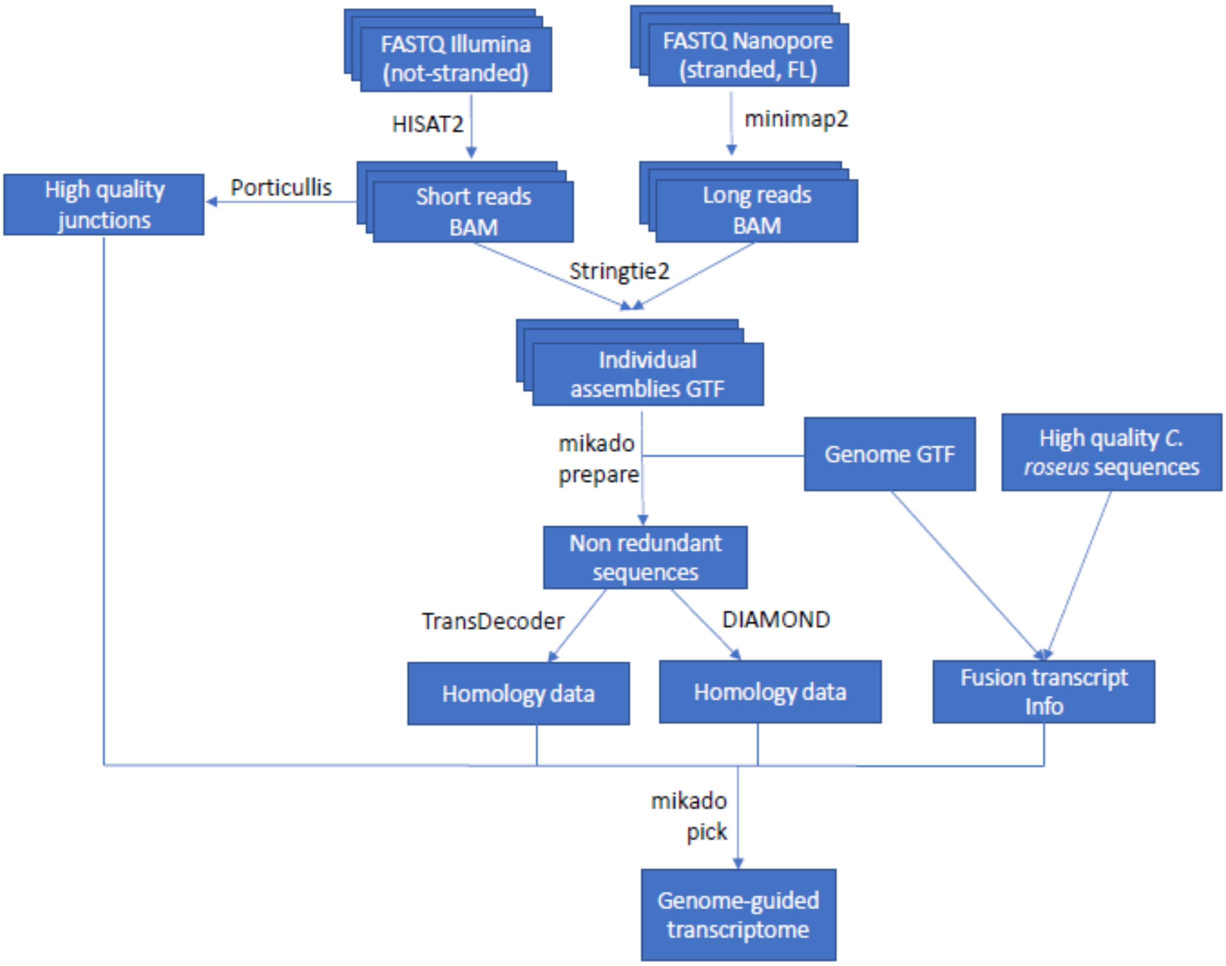

Supplementary Fig. S4. Summary of the approach used for genome-guided transcriptome assembly. Blue boxes represent data and arrows represent the software used. Briefly, the reads were aligned using HISAT2 and minimap2 (short and long read data respectively) and gene models were assembled using Stringtie. Mikado was used to filter, merge and refine gene models using information from high quality junctions (derived from Portcullis), open reading frame coordinates (TransDecoder) and homology data (DIAMOND). Furthermore, fusion transcripts were filtered by mapping genes to high quality *C. roseus* sequences from CD97 (Dugé de Bernonville *et al.*, 2015b) and the GenBank. The final set of transcripts was obtained using Mikado pick.

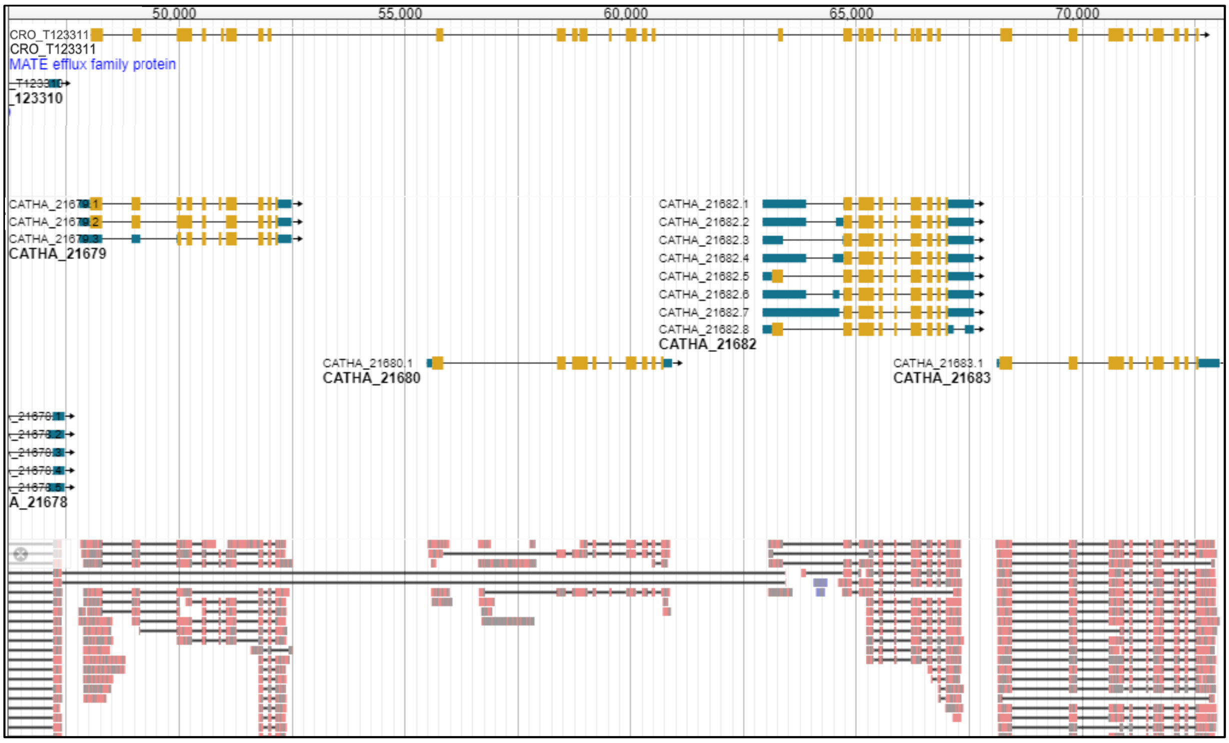

Supplementary Fig. S5. Screenshot obtained with the genome browser Jbrowse (jbrowse.org) depicting an example of an erroneous gene-model of a MATE protein present in the reference genome annotation (CRO_T123311). Genes with the prefix "CATHA" are models assembled in the scope of this work. Nanopore data is represented in rose. The data clearly shows that the genome reference gene model is actually the result of the tandem merging of several protein-coding genes.

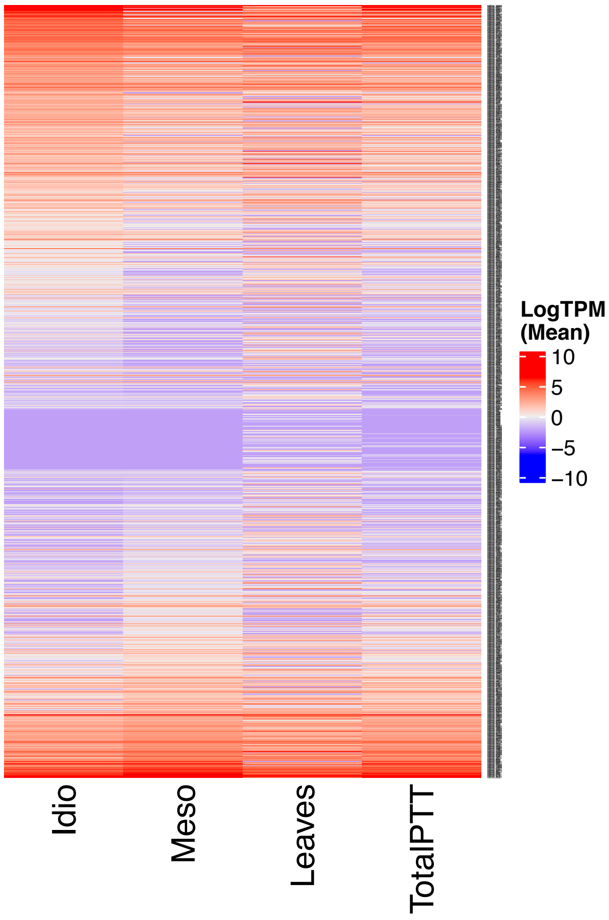

Supplementary Fig. S6. Gene expression heat map of the 1,128 novel gene loci identified in IDIO+ in comparison with the *C.roseus* genome v2 reference annotation, in the different tissue and cell samples involved in the isolation of idioblasts. The data and gene IDs are included in Supplementary Table S1. 567 of the new genes are expressed in idioblasts. TPM - transcripts per million, calculated as described in Materials and Methods.

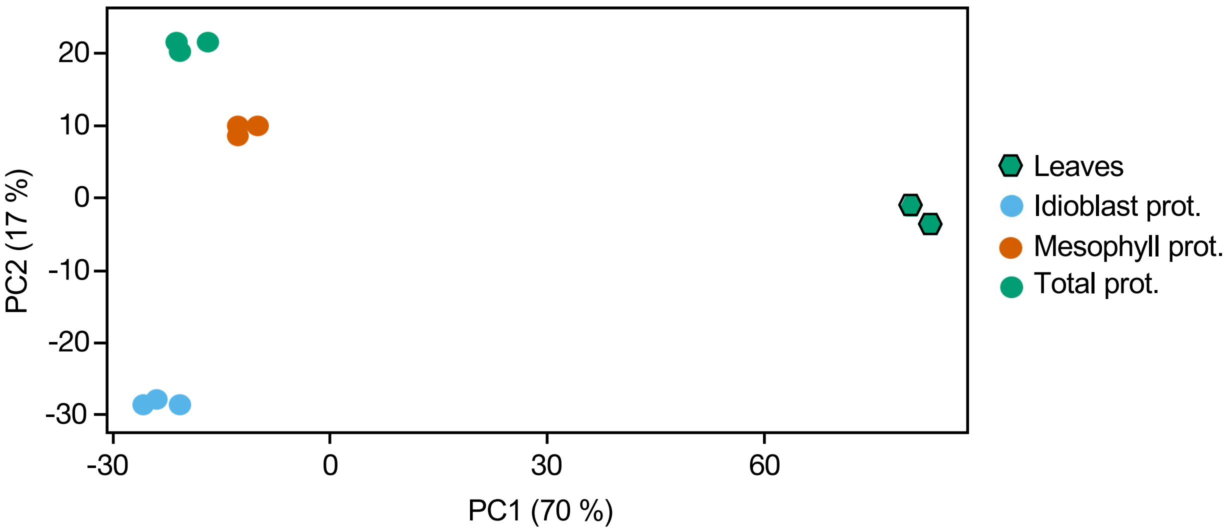

Supplementary Fig. S7. Principal component analysis (PCA) of the transcriptomic datasets generated for the different tissue and cell samples involved in the isolation of idioblasts (Fig. 2C, upper part). Analysis was based on the regularized log transformation of normalized read counts per gene. All the different protoplast populations are clearly isolated from each other, away from leaves, with total protoplasts being closer to common mesophyll cells than to idioblasts, as expected, due to the much higher abundance of common mesophyll cells compared with idioblasts.

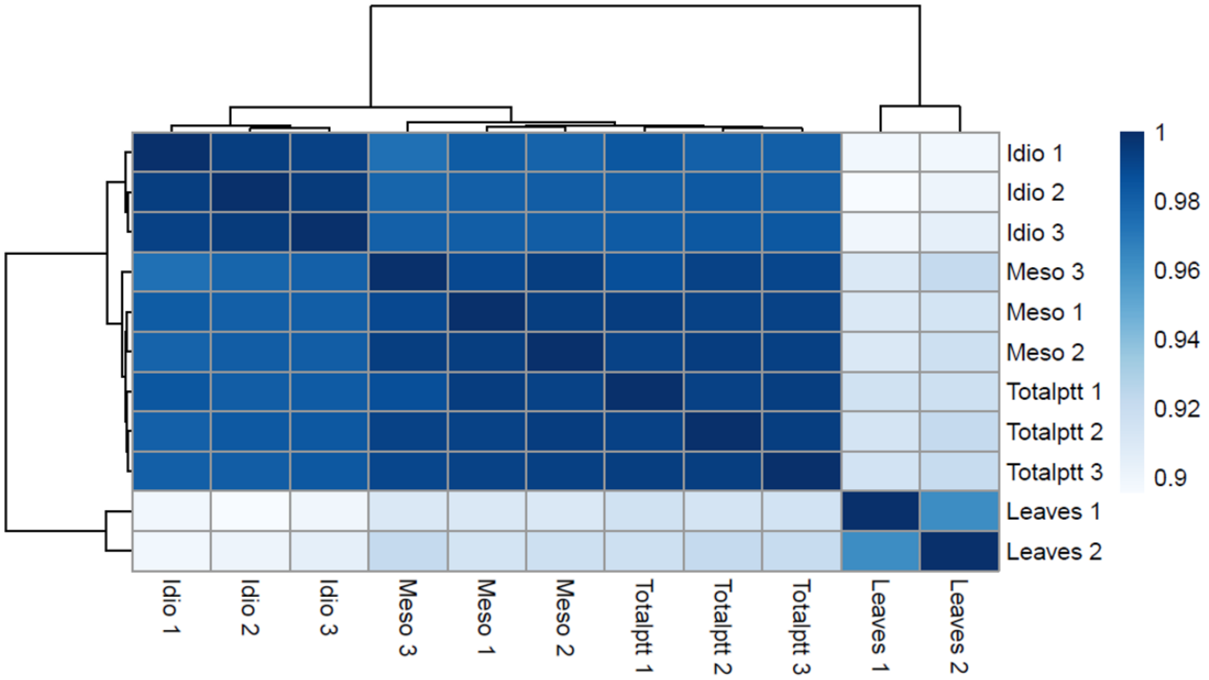

Supplementary Fig. S8. Hierarchical clustering of the different tissue and cell samples involved in the isolation of idioblasts, based on the regularized log transformation of normalized read counts per gene. Idioblast and leaf samples are well resolved, while total and mesophyll protoplast populations are not completely resolved. This may be explained by the fact that mesophyll cells are overrepresented in total protoplasts.

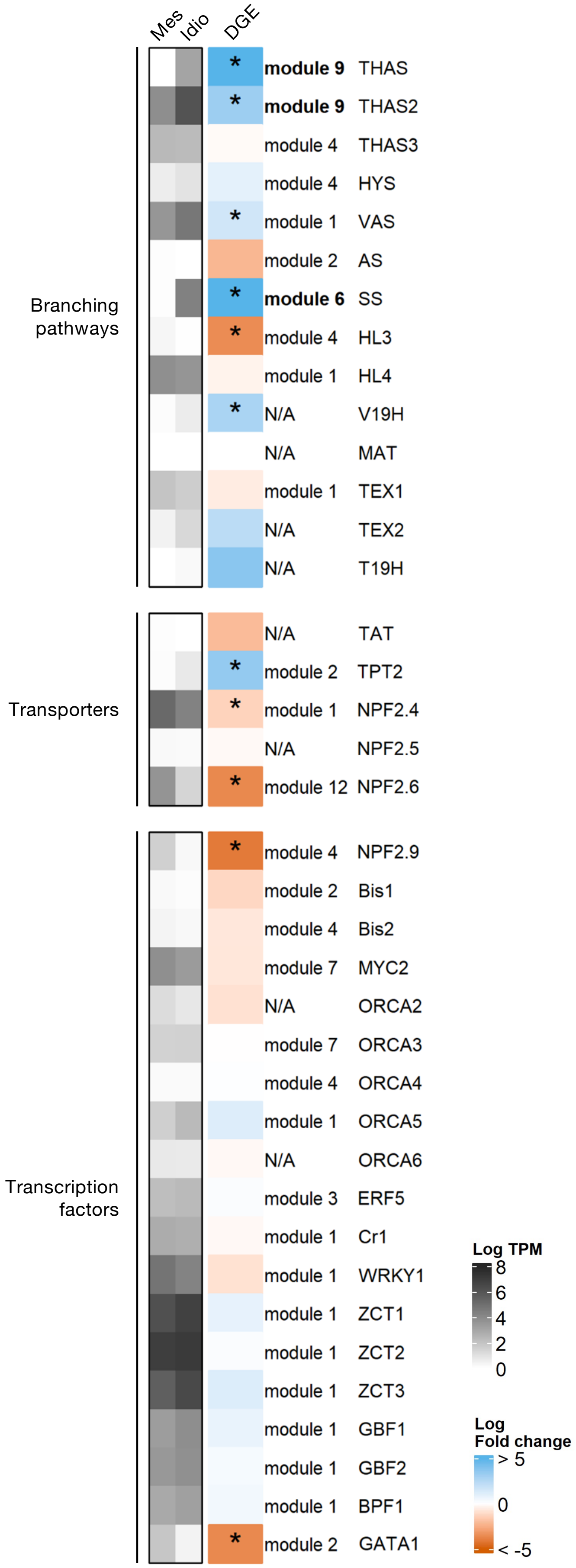

Supplementary Fig. S9. Expression analysis of genes that have been implicated in MIA branching pathways (Fig. 1), in MIA transmembrane transport and in transcriptional regulation of the MIA pathway. Heatmap grey colours represent the mean log2 TPM of three independent biological samples of common mesophyll protoplasts (Mes) and idioblast protoplasts (Idio). DGE, differential gene expression, represents the log2 fold change for each gene, with blue and red orange representing respectively overexpression and underexpression in idioblast protoplasts compared with common mesophyll protoplasts. Significant differential expression is marked with *. Abbreviations: THAS - tetrahydroalstonine synthase; HYS - heteroyohimbine synthase; VAS - vitrosamine synthase; AS - alstonine synthase; SS – serpentine synthase; HL3 - hydrolase 3; HL4 - hydrolase 4; V19H - vincadifformine 19-hydroxylase; MAT - minovincinine-O-acetytransferase; TEX - tabersonine 6,7-epoxidase; T19H - taberstonine 19-hydroxylase; TAT - tabersonine-19 O-acetyltransferase; NPF - nitrate/peptide family; Bis - bHLH iridoid synthesis, MYC - myelocytomatosis; ORCA - octadecanoid-responsive Catharanthus AP2-domain; ERF - ethylene responsive factor; CR1 - Catharanthus roseus protein 1; ZCT - Zinc finger Catharanthus transcription factor; GBF - G-box-binding factors; BPF1 - box P-binding factor-1; GATA1 - GATA binding protein 1. Gene IDs in Table S9; TPM values in Dataset S4; DGE in Supplementary Table S2.

P450s

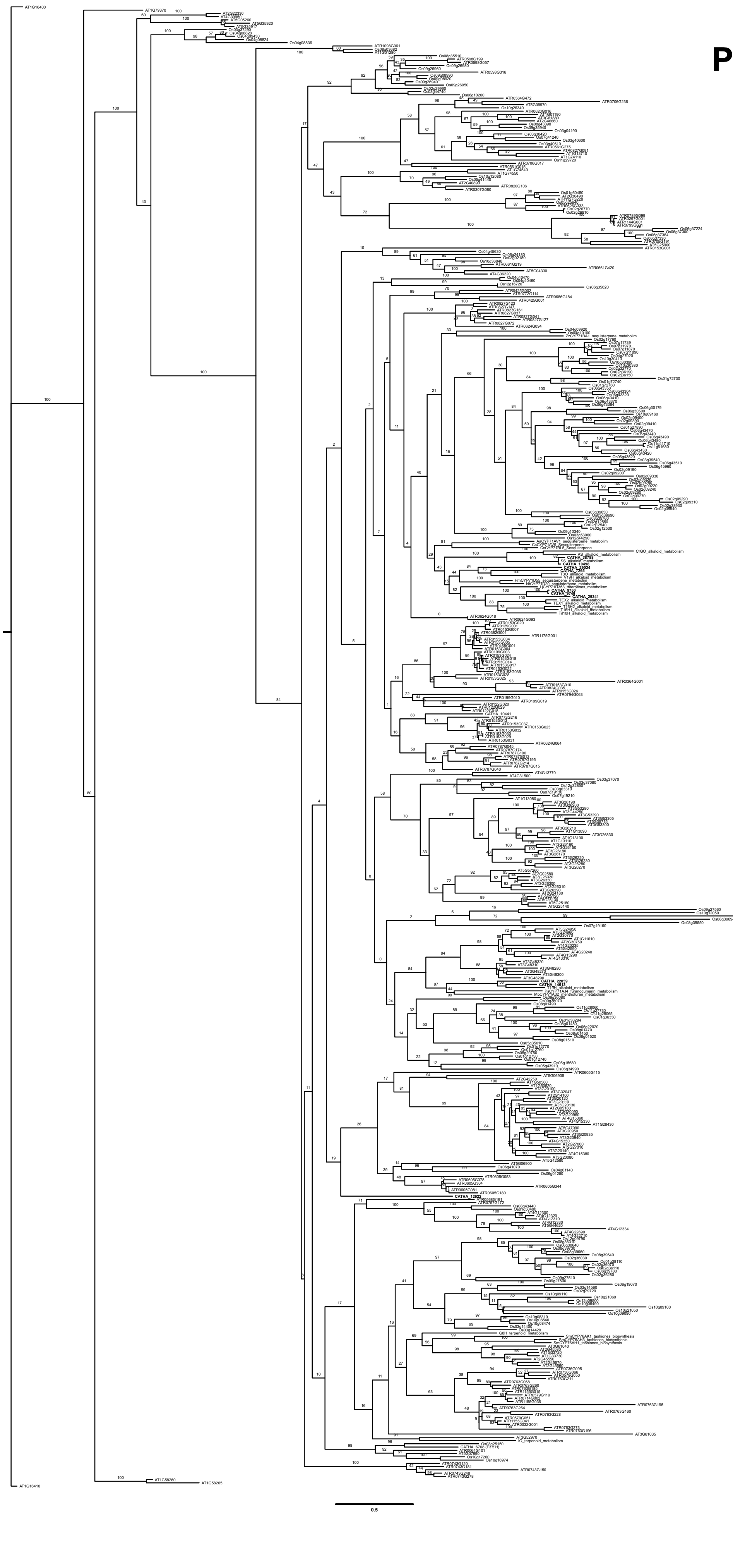

Supplementary Figure S10. Phylogenetic analysis of cytochrome P450s from clan 71 upregulated in *C. roseus* idioblasts. P450 proteins from clan 71 of *Arabidopsis thaliana* (AT), *Oryza sativa* (Os) and *Amborella trichopoda* (ATR), together with clan 71 P450s with known roles in specialised metabolism from other plant origins (Supplementary Table S12) were used in the phylogenetic analysis. Amino acid sequences were aligned using MAFFT (Katoh and Standley 2013), and the maximum likelihood method was used to construct a phylogenetic tree with 1000 bootstrap replications. Numbers at the nodes represent the bootstrap value (maximum 100) supporting the respective clade. CATHA – IDIO+ gene IDs. Species: Aa, *Artemisia annua*; Cc, *Cynara cardunculus*; Hm, *Hyoscyamus muticus;* Lj, *Lotus japonicus*; Mp, *Mentha piperita*; Nt, *Nicotiana tabacum*; Ps, *Pastinaca sativa;* Sm, *Salvia miltiorrhiza;* Ti, *Tabernanthe iboga*; Zz, *Zingiber zerumbet*. Enzymes: AS, alstonine synthase; CYP, cytochrome P450; G8H, geraniol 8-hydroxylase; GO, geissoschizine oxidase; I10H, ibogamine 10-hydroxylase; IO, iridoid oxidase; SS, serpentine synthase; T3O, tabersonine 3-oxygenase; T16H, tabersonine 16-hydroxylase; TEX, tabersonine epoxidase; V19H, vincadifformine-19-hydroxylase.

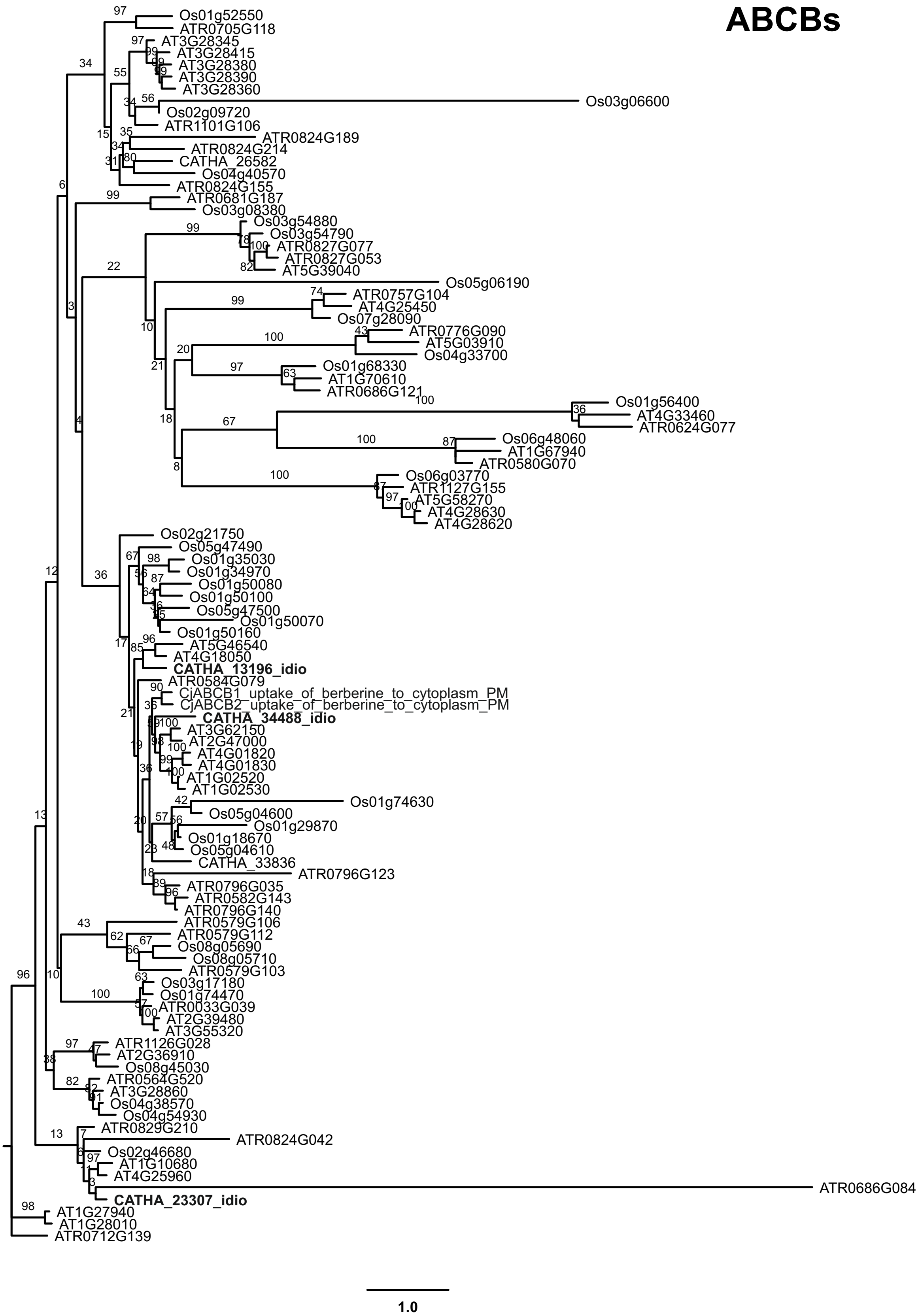
ABCBs
Os01g52550
ATR0705G118
AT3G28345
AT3G28415
AT3G28380
AT3G28390
AT3G28360
Os03g06600
Os02g09720
ATR1101G106
ATR0824G189
ATR0824G214
CATHA_26582
Os04g40570
ATR0824G155
ATR0681G187
Os03g08380
Os03g54880
Os03g54790
ATR0827G077
ATR0827G053
AT5G39040
Os05g06190
ATR0757G104
AT4G25450
Os07g28090
ATR0776G090
AT5G03910
Os04g33700
Os01g68330
AT1G70610
ATR0686G121
Os01g56400
AT4G33460
ATR0624G077
Os06g48060
AT1G67940
ATR0580G070
Os06g03770
ATR1127G155
AT5G58270
AT4G28630
AT4G28620
Os02g21750
Os05g47490
Os01g35030
Os01g34970
Os01g50080
Os01g50100
Os05g47500
Os01g50070
Os01g50160
AT5G46540
AT4G18050
CATHA_13196_idio
ATR0584G079
CjABCB1_uptake_of_berberine_to_cytoplasm_PM
CjABCB2_uptake_of_berberine_to_cytoplasm_PM
CATHA_34488_idio
AT3G62150
AT2G47000
AT4G01820
AT4G01830
AT1G02520
AT1G02530
Os01g74630
Os05g04600
Os01g29870
Os01g18670
Os05g04610
CATHA_33836
ATR0796G123
ATR0796G035
ATR0582G143
ATR0796G140
ATR0579G106
ATR0579G112
Os08g05690
Os08g05710
ATR0579G103
Os03g17180
Os01g74470
ATR0033G039
AT2G39480
AT3G55320
ATR1126G028
AT2G36910
Os08g45030
ATR0564G520
AT3G28860
Os04g38570
Os04g54930
ATR0829G210
ATR0824G042
Os02g46680
AT1G10680
AT4G25960
ATR0686G084
CATHA_23307_idio
AT1G27940
AT1G28010
ATR0712G139
1.0

Supplementary Fig. S11. Phylogenetic analysis of ABCB transmembrane transporters upregulated in *C. roseus* idioblasts. ABCB proteins from *Arabidopsis thaliana* (AT), *Oryza sativa* (Os), *Amborella trichopoda* (ATR) and selected ABCBs with known roles in specialized metabolism (Supplementary Table S14) were used in the phylogenetic analysis. Amino acid sequences were aligned using MAFFT (Katoh and Standley 2013), and the maximum likelihood method was used to construct a phylogenetic tree with 1000 bootstrap replications. Numbers at the nodes represent the bootstrap value (maximum 100) supporting the respective clade. CATHA – IDIO+ gene IDs. Cj, *Coptis japonica.*

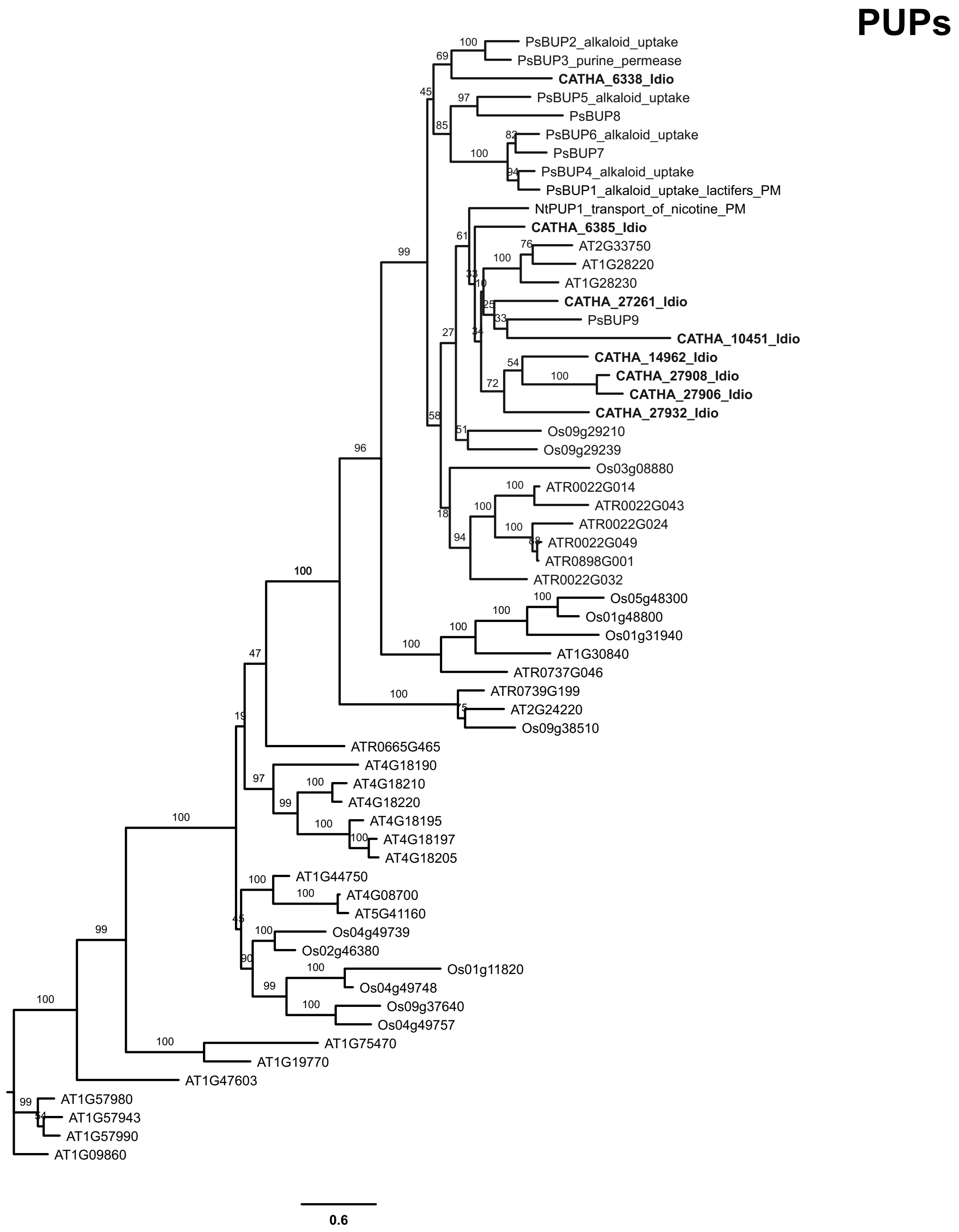
PUPs
PsBUP2_alkaloid_uptake
PsBUP3_purine_permease
CATHA_6338_Idio
PsBUP5_alkaloid_uptake
PsBUP8
PsBUP6_alkaloid_uptake
PsBUP7
PsBUP4_alkaloid_uptake
PsBUP1_alkaloid_uptake_lactifers_PM
NtPUP1_transport_of_nicotine_PM
CATHA_6385_Idio
AT2G33750
AT1G28220
AT1G28230
CATHA_27261_Idio
PsBUP9
CATHA_10451_Idio
CATHA_14962_Idio
CATHA_27908_Idio
CATHA_27906_Idio
CATHA_27932_Idio
Os09g29210
Os09g29239
Os03g08880
ATR0022G014
ATR0022G043
ATR0022G024
ATR0022G049
ATR0898G001
ATR0022G032
Os05g48300
Os01g48800
Os01g31940
AT1G30840
ATR0737G046
ATR0739G199
AT2G24220
Os09g38510
ATR0665G465
AT4G18190
AT4G18210
AT4G18220
AT4G18195
AT4G18197
AT4G18205
AT1G44750
AT4G08700
AT5G41160
Os04g49739
Os02g46380
Os01g11820
Os04g49748
Os09g37640
Os04g49757
AT1G75470
AT1G19770
AT1G47603
AT1G57980
AT1G57943
AT1G57990
AT1G09860
0.6

Supplementary Fig. S12. Phylogenetic analysis of PUP transmembrane transporters upregulated in *C. roseus* idioblasts. PUP proteins from *Arabidopsis thaliana* (AT), *Oryza sativa* (Os), *Amborella trichopoda* (ATR) and selected PUPs with known roles in specialized metabolism (Supplementary Table S15) were used in the phylogenetic analysis. Amino acid sequences were aligned using MAFFT (Katoh and Standley 2013), and the maximum likelihood method was used to construct a phylogenetic tree with 1000 bootstrap replications. Numbers at the nodes represent the bootstrap value (maximum 100) supporting the respective clade. CATHA – IDIO+ gene IDs. Nt, *Nicotiana tabacum*; Ps, *Papaver somniferum.*

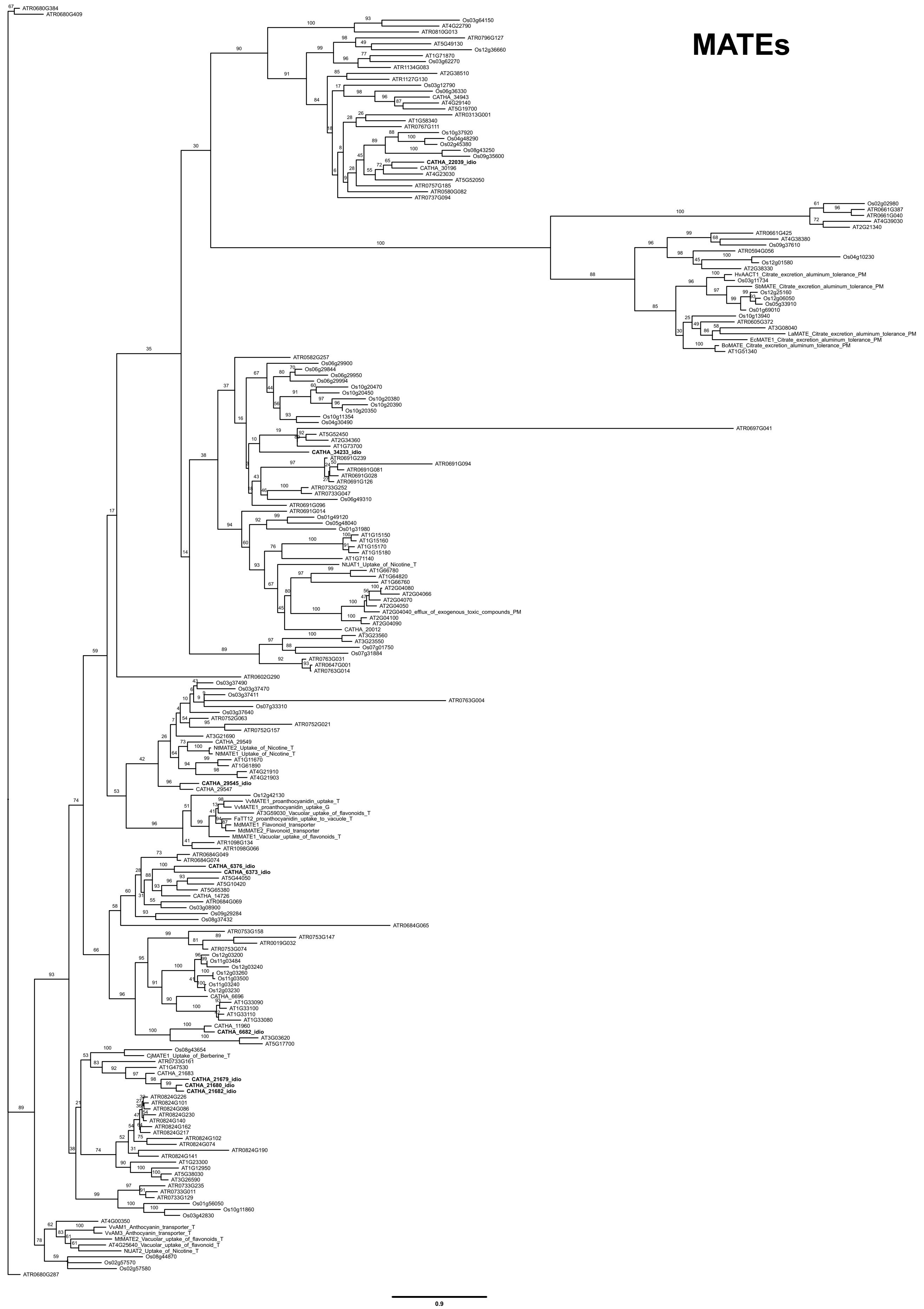
MATEs
ATR0680G384
ATR0680G409
Os03g64150
AT4G22790
ATR0810G013
ATR0796G127
AT5G49130
Os12g36660
AT1G71870
Os03g62270
ATR1134G083
AT2G38510
ATR1127G130
Os03g12790
Os06g36330
CATHA_34943
AT4G29140
AT5G19700
ATR0313G001
AT1G58340
ATR0767G111
Os10g37920
Os04g48290
Os02g45380
Os08g43250
Os09g35600
CATHA_22039_idio
CATHA_30196
AT4G23030
AT5G52050
ATR0757G185
ATR0580G082
ATR0737G094
Os02g02980
ATR0661G387
ATR0661G040
AT4G39030
AT2G21340
ATR0661G425
AT4G38380
Os09g37610
ATR0594G056
Os04g10230
Os12g01580
AT2G38330
HvAACT1_Citrate_excretion_aluminum_tolerance_PM
Os03g11734
SbMATE_Citrate_excretion_aluminum_tolerance_PM
Os12g25160
Os12g06050
Os05g33910
Os01g69010
Os10g13940
ATR0605G372
AT3G08040
LaMATE_Citrate_excretion_aluminum_tolerance_PM
EcMATE1_Citrate_excretion_aluminum_tolerance_PM
BoMATE_Citrate_excretion_aluminum_tolerance_PM
AT1G51340
ATR0582G257
Os06g29900
Os06g29844
Os06g29950
Os06g29994
Os10g20470
Os10g20450
Os10g20380
Os10g20390
Os10g20350
Os10g11354
Os04g30490
ATR0697G041
AT5G52450
AT2G34360
AT1G73700
CATHA_34233_idio
ATR0691G239
ATR0691G094
ATR0691G081
ATR0691G028
ATR0691G126
ATR0733G252
ATR0733G047
Os06g49310
ATR0691G096
ATR0691G014
Os01g49120
Os05g48040
Os01g31980
AT1G15150
AT1G15160
AT1G15170
AT1G15180
AT1G71140
NtJAT1_Uptake_of_Nicotine_T
AT1G66780
AT1G64820
AT1G66760
AT2G04080
AT2G04066
AT2G04070
AT2G04050
AT2G04040_efflux_of_exogenous_toxic_compounds_PM
AT2G04100
AT2G04090
CATHA_20012
AT3G23560
AT3G23550
Os07g01750
Os07g31884
ATR0763G031
ATR0647G001
ATR0763G014
ATR0602G290
Os03g37490
Os03g37470
Os03g37411
ATR0763G004
Os07g33310
Os03g37640
ATR0752G063
ATR0752G021
ATR0752G157
AT3G21690
CATHA_29549
NtMATE2_Uptake_of_Nicotine_T
NtMATE1_Uptake_of_Nicotine_T
AT1G11670
AT1G61890
AT4G21910
AT4G21903
CATHA_29545_idio
CATHA_29547
Os12g42130
VvMATE1_proanthocyanidin_uptake_T
VvMATE1_proanthocyanidin_uptake_G
AT3G59030_Vacuolar_uptake_of_flavonoids_T
FaTT12_proanthocyanidin_uptake_to_vacuole_T
MdMATE1_Flavonoid_transporter
MdMATE2_Flavonoid_transporter
MtMATE1_Vacuolar_uptake_of_flavonoids_T
ATR1098G134
ATR1098G066
ATR0684G049
ATR0684G074
CATHA_6376_idio
CATHA_6373_idio
AT5G44050
AT5G10420
AT5G65380
CATHA_14726
ATR0684G069
Os03g08900
Os09g29284
Os08g37432
ATR0684G065
ATR0753G158
ATR0753G147
ATR0019G032
ATR0753G074
Os12g03200
Os11g03484
Os12g03240
Os12g03260
Os11g03500
Os11g03240
Os12g03230
CATHA_6696
AT1G33090
AT1G33100
AT1G33110
AT1G33080
CATHA_11960
CATHA_6682_idio
AT3G03620
AT5G17700
Os08g43654
CjMATE1_Uptake_of_Berberine_T
ATR0733G161
AT1G47530
CATHA_21683
CATHA_21679_idio
CATHA_21680_idio
CATHA_21682_idio
ATR0824G226
ATR0824G101
ATR0824G086
ATR0824G230
ATR0824G140
ATR0824G162
ATR0824G217
ATR0824G102
ATR0824G074
ATR0824G190
ATR0824G141
AT1G23300
AT1G12950
AT5G38030
AT3G26590
ATR0733G235
ATR0733G011
ATR0733G129
Os01g56050
Os10g11860
Os03g42830
AT4G00350
VvAM1_Anthocyanin_transporter_T
VvAM3_Anthocyanin_transporter_T
MtMATE2_Vacuolar_uptake_of_flavonoids_T
AT4G25640_Vacuolar_uptake_of_flavonoid_T
NtJAT2_Uptake_of_Nicotine_T
Os08g44870
Os02g57570
Os02g57580
ATR0680G287
0.9

Supplementary Fig. S13. Phylogenetic analysis of MATE transmembrane transporters upregulated in *C. roseus* idioblasts. MATE proteins from *Arabidopsis thaliana* (AT), *Oryza sativa* (Os), *Amborella trichopoda* (ATR) and selected MATEs with known roles in specialized metabolism (Supplementary Table S16) were used in the phylogenetic analysis. Amino acid sequences were aligned using MAFFT (Katoh and Standley 2013), and the maximum likelihood method was used to construct a phylogenetic tree with 1000 bootstrap replications. Numbers at the nodes represent the bootstrap value (maximum 100) supporting the respective clade. CATHA – IDIO+ gene IDs. Species: Bo, *Brassica oleracea*; Ec, *Eucalyptus camaldulensis*; Cj, Coptis japonica; Fa, *Fragaria vesca*; Hv, *Hordeum vulgare*; La, *Lupinus albus*; Md, Malus domestica; Mt, *Medicago truncatula*; Nt, *Nicotiana tabacum*; Sb, *Sorghum bicolor*; Md, *Malus domestica*; Vv, *Vitis vinifera*. Transporters: AM, anthoMATE; JAT, jasmonate-inducible alkaloid transporter : TT, transparent testa.
